# High‐Entropy PdRhFeCoMo Metallene With High C1 Selectivity and Anti‐Poisoning Ability for Ethanol Electrooxidation

**DOI:** 10.1002/advs.202409109

**Published:** 2024-11-19

**Authors:** Xiaohong Tan, Chenhui Wang, Jiarui Wang, Peng Wang, Yuhang Xiao, Yingying Guo, Jianpo Chen, Weidong He, Yan Li, Hao Cui, Chengxin Wang

**Affiliations:** ^1^ School of Materials Science and Engineering Sun Yat‐sen University Guangzhou 510275 China

**Keywords:** anti‐poisoning ability, elevated C1 selectivity, ethanol oxidation reaction, flexible solid‐state fuel cell, high‐entropy metallene

## Abstract

The urgent demand for designing highly efficient electrocatalysts for ethanol oxidation reaction (EOR) with elevated C1 selectivity, robust anti‐poisoning capability, and high mass activity presents a formidable challenge. Herein, a novel two‐dimentional (2D) high‐entropy PdRhFeCoMo metallene (PdRhFeCoMo HEM) electrocatalyst is successfully synthesized via a mild one‐step solvothermal method. The PdRhFeCoMo HEM, characterized by intentionally designed multi‐metallic ensembles and ultra‐thin graphene‐like structures, delivers an impressive mass activity of 7.47 A mg_Pd+Rh_^−1^ and specific activity of 25.5 mA cm^−2^. Furthermore, it can retain a mass activity of 0.56 A mg_Pd+Rh_^−1^ after undergoing 20000 s of continuous testing, demonstrating outstanding resistance to poisoning. More significantly, the PdRhFeCoMo HEM demonstrates an elevated capacity for C─C bond cleavage with a superior C1 selectivity of up to 84.12%. In situ spectroscopy analysis, combined with theoretical calculations, reveals that the deliberate design of components and structures effectively regulate the electronic properties of the Pd site, thereby enhancing the adsorption of reactant and reducing the reaction barrier of the C1 pathway. Finally, a flexible solid‐state ethanol fuel cell assembled by PdRhFeCoMo HEM presents a maximum power density of 20.1 mW cm^−2^ and can operate continuously by repeatedly adding ethanol fuel.

## Graphical abstract

A novel 2D high‐entropy PdRhFeCoMo metallene is synthesized via a mild one‐step solvothermal method as an excellent EOR catalyst. The deliberate design of morphology, composition, and structure for the PdRhFeCoMo HEM effectively regulate the electronic properties of the Pd site, thereby enhancing the adsorption of reactant, resistance to poisoning and reducing the energy barrier for C─C bond breaking.

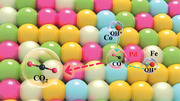

## 1 Introduction

Fuel cells (FCs) have garnered significant attention as energy storage and conversion devices due to their low corrosion, well‐established infrastructure, and industrial maturity.^[^ ^1^, ^2^ ^]^ In comparison to other fuels, ethanol offers a significantly higher theoretical volumetric energy density (6.28 kWh L^−1^) than hydrogen fuel (1.3 kWh L^−1^) and methanol fuel (4.82 kWh L^−1^), making EOR fuel cells a key area of research focus.^[^ ^3^, ^4^ ^]^ However, the alkaline EOR process involves multiple electron pathways with sluggish kinetics and the formation of severe intermediate by‐products. Up until now, noble metals such as platinum (Pt) and palladium (Pd) have been widely regarded as the most favorable electrocatalysts for EOR.^[^ ^5^, ^6^, ^7^ ^]^ Unfortunately, noble metals Pt and Pd are susceptible to poisoning by CO, an intermediate produced during the alkaline EOR process, leading to a substantial decrease in catalytic activity.^[^ ^8^, ^9^, ^10^ ^]^ The EOR follows a so‐called dual‐pathway mechanism: the complete oxidation of ethanol to CO_2_ with 12 electrons transfer (C1 pathway) and the incomplete oxidation of ethanol to acetic acid or acetate with 4 electrons transfer (C2 pathway). Among them, C1 pathway with more electron transfer apparently contributes to the desired higher output current and thus elevated power density. However, in the majority of Pt‐ and Pd‐based EOR catalysts, the preferred C1 pathway is greatly hindered due to their weak C─C bond cleavage.^[^ ^11^, ^12^, ^13^ ^]^ In addition, the high cost of noble metal catalysts further limits their large‐scale application in EOR. Therefore, there is an urgent demand to design and synthesize EOR catalysts with high C1 selectivity, strong anti‐poisoning ability, and outstanding mass activity (noble metals).^[^ ^14^ ^]^

High‐entropy alloys (HEAs), characterized by the incorporation of five or more metal components with concentrations ranging from 5 to 35 at % in single‐phase solid solutions, have recently been considered as potential catalyst materials due to their unique structure and compositional advantages.^[^ ^15^, ^16^, ^17^ ^]^ The alloying effect among multiple metals in HEAs gives rise to a mechanically robust structure that is highly resistant to both acidic and alkaline environments.^[^ ^18^ ^]^ Furthermore, the incorporation of cost‐effective, oxygen‐affine metals into HEAs not only offers potential cost savings for the catalyst but also enhances its resistance to poisoning by leveraging the synergistic effects among metals.^[^ ^19^ ^]^ Recently, there have been reports that high entropy alloys have superior EOR properties in comparison to binary/ternary alloys.^[^ ^4^, ^8^, ^18^ ^]^ Despite the significant progress made in EOR using noble‐metal‐based HEAs, the current majority of HEAs catalysts face challenges such as underutilization of catalytic active sites due to alloying effects, excessive usage of noble metals, and sluggish diffusion of toxic intermediates.^[^ ^19^ ^]^ Additionally, the synthesis of HEAs often involves the harsh conditions (i.e., high temperatures and expensive synthesis equipment). Therefore, the rational design of HEAs for EOR poses an alluring yet challenging task, involving the regulation of active site‐electrolyte interaction, optimization of noble metal active site utilization, and facilitation of removal of toxic intermediate.^[^ ^20^ ^]^

Metallene, a graphene‐like metastable 2D material with atomic‐level thickness, exhibits an ultra‐high surface area, offering a promising avenue to optimize the exposure of catalytic active sites.^[^ ^21^, ^22^, ^23^ ^]^ Furthermore, the curvature of metallenes caused by the minimization of surface free energy can also induce lattice strain, optimizing the adsorption behavior of reaction intermediates.^[^ ^24^ ^]^ As a result of this characteristic, metallene has attracted widespread attention in various catalysis fields.^[^ ^23^, ^25^, ^26^, ^27^ ^]^ Recently, metallene‐based materials has been explored as a promising electrocatalyst for enhancing EOR performance.^[^ ^2^, ^24^, ^28^ ^]^ However, the metallenes reported so far mainly consist of binary or ternary metals, and the application of HEAs metallenes containing five or more metal species in EOR remains relatively rare.

By integrating the distinctive morphological advantages of 2D metallenes with the pronounced alloy effects of HEAs, we rationally designed an ultrathin 2D PdRhFeCoMo HEM as a highly efficient EOR catalyst. Notably, this pioneering study marks the first‐ever application of high‐entropy metallene catalysts in the field of EOR. Herein, Pd was selected as the active metal due to its promising activity and stability for EOR under alkaline conditions. Concurrently, cost‐effective metals Fe and Co were carefully selected because they not only can form solid solutions with Pd, reducing the amount of noble metals, but also can adsorb OH^*^ and O^*^ at low voltages, effectively facilitating the removal of toxic intermediates.^[^ ^4^ ^]^ Additionally, the introduction of Rh strengthens the C─C bond cleavage of metallene,^[^ ^29^ ^]^ while the presence of Mo played a crucial role in assisting the formation of ultrathin metallene structures in Pd‐based catalysts.^[^ ^24^ ^]^ The deliberate design of morphology, composition, and structure is aimed at optimizing both the economic feasibility and catalytic performance of EOR catalysts. As expected, the ultrathin high‐entropy metallene with a thickness of ≈1.0 nm exhibited excellent mass activity (7.47 A mg_Pd+Rh_^−1^, 9.96 A mg_Pd_^−1^), outstanding resistance to poisoning (retaining a mass activity of 0.56 A mg_Pd+Rh_^−1^ after 20 000 s of chronoamperometry test), and remarkable C─C bond cleavage ability (possible C1 pathway accounts for 84.12%). Theoretical calculations demonstrate that the introduction of multiple metals can tune the *d*‐band center of surface Pd atoms to upshift toward the Fermi energy, enhancing the adsorption of ethanol and reducing the energy barrier for C─C bond breaking. When assembled in a flexible solid‐state ethanol fuel cell, the PdRhFeCoMo HEM based‐cell demonstrated a maximum power density of 20.1 mW cm^−2^ and was capable of operating at various bending angles, highlighting the excellent EOR performance of PdRhFeCoMo HEM and promised potential in the application for energy device.

## 2 Results and Discussion

**Figure 1:**
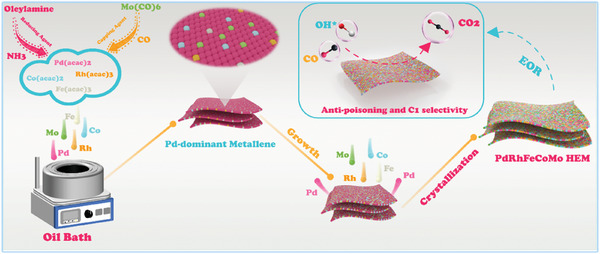
Schematic illustration of the synthesis of PdRhFeCoMo HEM.

In the present experiment, acetylacetone palladium, acetylacetone rhodium, acetylacetone iron, and acetylacetone cobalt were employed as metal precursors. Molybdenum hexacarbonyl serves a dual purpose in the experiment. It not only acts as a metal source but can also be decomposed at high temperatures to produce CO, which serves as a capping agent. The lack of molybdenum hexacarbonyl inhibits the formation of ultrathin metallenes (Figure S1, Supporting Information). Additionally, ammonia derived from the heating decomposition of oleoamine could act as a reducing agent.^[^ ^22^ ^]^ By observing the products at different reaction times (Figures S2–S4, Supporting Information), we have proposed the following growth mechanism for ultra‐thin PdRhFeCoMo HEM, as shown in **Figure** **1**. In the initial stage, Pd metal is preferentially reduced and forms a metallene morphology in the presence of the capping agent, and a small amount of other metals are also reduced and implanted into metallene. Then, the metallene grows along the lateral direction, and undergoes compositional changes (reducing the amount of Pd) through galvanic reactions between Pd atoms and other metal ions. As the reaction continued, the high‐entropy alloy metallene forms with the concentration of each component in the range of 5–35 at %. Notably, in the absence of Pd, no metallene formation was observed (RhFeCoMo, Figure S5, Supporting Information), thereby confirming the proposed growth mechanism. Finally, by reducing the number of metal precursors, medium‐entropy metallene (MEM, quaternary PdFeCoMo) and low‐entropy metallene (LEM, binary PdMo) were also prepared in the same way as control samples (Figures S6 and S7, Supporting Information, details in the Experimental Section).

**Figure 2:**
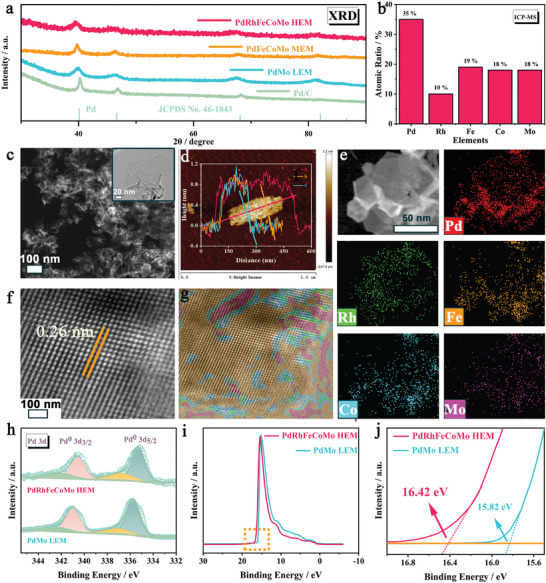
Morphology and structure characterization of metallenes a) XRD patterns. b) ICP‐MS result of atomic ratio. c) The dark‐field STEM image. Inset of TEM image. d) AFM image and corresponding height profile across a high‐entropy metallene. e) STEM and corresponding elemental mapping of Pd, Rh, Fe, Co, and Mo. f) HRTEM image. g) Geometric phase analysis (GPA) for the lattice strain distribution taken from the selected areas in HRTEM image. h) XPS spectra of Pd *3d* of PdRhFeCoMo HEM and PdMo LEM. i–j) UPS spectra of PdRhFeCoMo HEM and PdMo LEM.

X‐ray powder diffraction (XRD) characterization was conducted on all samples (**Figure** **2**a). All XRD patterns closely resemble those of Pd, exhibiting three prominent peaks at 40.1°, 46.6°, and 68.1°, corresponding to the (111), (200), and (220) facets of the face‐centered cubic (*fcc*) structure.^[^ ^30^ ^]^ Notably, the XRD peaks of all metallenes exhibit a significant leftward shift compared to the Pd/C sample, with overlapping peak positions. Since Pd has a larger atomic radius than the other metals (Rh, Fe, and Co), this uniform peak shift observed across these different metallenes can be attributed to the inherent tensile strain induced by their highly curved morphology.^[^ ^28^ ^]^ The atomic ratio of Pd/Rh/Fe/Co/Mo in PdRhFeCoMo HEM is determined to be 35/10/19/18/18 (ICP‐MS), consistent with the near‐equiatomic composition characteristic of high‐entropy alloys (Figure 2b). Transmission electron microscopy (TEM) and high angle annular dark field scanning transmission electron microscopy (HAADF) images reveal that the PdRhFeCoMo HEM possesses an ultrathin, wrinkled, 2D graphene‐like nanosheet structure with uniform dispersion, as shown in Figure 2c and HAADF. Atomic force microscopy (AFM) images further confirm the ultrathin nature of the sample with a thickness of ≈1.0 nm, equivalent to 5–6 atomic layers Figure 2d; Figure S8 and Table S1 (Supporting Information). HAADF‐STEM energy‐dispersive X‐ray (EDX) elemental mappings confirm the presence of five elements‐Pd, Rh, Fe, Co, and Mo‐in the metallene (Figure 2e). Besides, PdFeCoMo MEM, and PdMo LEM also demonstrate ultrathin 2D metallene structures with the designed elements (Figures S6 and S7, Supporting Information). High‐resolution TEM (HRTEM, Figure 2f) image clearly reveals the orderly arrangement of atoms in the PdRhFeCoMo HEM. A clear lattice fringe of 0.26 nm, corresponding to the (111) plane of *fcc* structure, significantly exceeds the standard Pd lattice spacing of 0.22 nm, indicating substantial tensile strain.^[^ ^31^, ^32^, ^33^ ^]^ The strain distribution was analyzed using the geometric phase analysis (GPA) method based on the individual atomic resolution HRTEM image, as shown in Figure 2g. Overall, the predominantly tensile strain distribution in PdRhFeCoMo HEM was verified by HRTEM, XRD, and GPA results.

X‐ray photoelectron spectroscopy (XPS) was conducted to deeply investigate the surface electronic structures of the different atoms. The XPS full spectrums indicate the coexistence of the designed elements on the surface of metallenes (Figure S9, Supporting Information). Pd and Rh existed mainly in a metallic state with slight oxidation. The transition metals Fe and Co predominantly exist in oxidation states of +2 and +3, as well as in metallic states with zero valence (Figure S10b,c, Supporting Information). Molybdenum is completely oxidized on the alloy surface, primarily manifesting in the oxidation states of Mo^4+^ and Mo^6+^ (Figure S10d, Supporting Information).^[^ ^34^, ^35^ ^]^ The two Pd *3d* peaks (335.3 and 340.56 eV, Figure 2h) in PdRhFeCoMo HEM exhibit negative shifts compared to the corresponding peaks observed in PdMo LEM (at 335.7 and 340.96 eV), indicating that Pd is in an electron‐rich state due to its higher electronegativity relative to Fe and Co (while Pd and Rh have similar electronegativities). The obtained results not only provide additional confirmation of the high‐entropy alloy state of PdRhFeCoMo HEM but also unveil the significant electronic interaction among its constituent elements. Based on the work function formula E_Φ_ = hv – |E_cutoff_ – E_F_|, the effective work functions of PdRhFeCoMo HEM and PdMo LEM are determined to be 4.78 and 5.38 eV, respectively (Figure 2i,j). Since the valence band of the high‐entropy alloy is primarily occupied by the *d* electrons of metals, the shift of the *d*‐band center can be positively correlated with the change in the position of the valence band top. Consequently, the reduction in the work function also indicates a decreased energy difference between the *d*‐band center and the vacuum energy level, which facilitates the adsorption of the intermediates and thereby boosts the kinetics of electrocatalytic reactions.^[^ ^36^ ^]^ This correlation will later be confirmed by the calculated *d*‐band center based on density functional theory (DFT) calculations.

**Figure 3:**
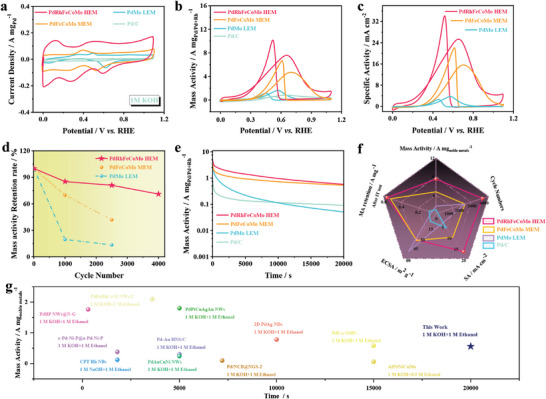
EOR performance evaluation of catalysts. CV curves of PdRhFeCoMo HEM, PdFeCoMo MEM, PdRhMo MEM, PdMo LEM, and commercial Pd/C recorded in a) 1 M KOH and b) 1 m KOH + 1 m CH_3_CH_2_OH electrolyte at a scan rate of 50 mV s^−1^. c) EOR Specific activity (normalized to the ECSAs). d) Cycling stability of metallene electrocatalysts. e) Long‐term chronoamperometry for EOR at 0.65 V versus RHE. f) Comparisions of EOR performance metrics. g) The *i*–*t* stability comparison of PdRhFeCoMo HEM with previously reported noble‐metal‐based catalysts.

Considering the inherent advantages resulting from the alloying effect among multiple metals and the ultra‐thin 2D structure, the PdRhFeCoMo HEM emerges as a highly promising catalyst for EOR. **Figure** **3**a shows the cyclic voltammogram (CV) curves of all the as‐prepared catalysts recorded in the 1 m KOH electrolyte solution. The underpotentially deposited hydrogen (H_UPD_) peaks observed at 0.2–0.4 V (vs reversible hydrogen electrode, RHE) in the cyclic voltammogram (CV) curves of PdRhFeCoMo HEM are significantly stronger compared to those of PdFeCoMo MEM, PdMo LEM, and Pd/C, indicating that PdRhFeCoMo HEM features more active surfaces and a more efficient utilization of noble metals.^[^ ^4^ ^]^ The peaks in the potential range of 0.4–0.8 V are attributed to the reduction of PdO during the negative scans, and the ECSA of each catalyst can be obtained by integrating this reduction peak area.^[^ ^37^, ^38^ ^]^ The ECSA of PdRhFeCoMo HEM was found to be 29.3 m^2^ g^−1^, which is similar to that of PdFeCoMo MEM (30.0 m^2^ g^−1^), PdMo LEM (42.4 m^2^ g^−1^), all of which were significantly higher than that of Pd/C (8.70 m^2^ g^−1^). This demonstrates that the 2D ultrathin structure can maximize the utilization of noble metal sites. In Pd‐deficient catalysts (RhFeCoMo), no current density for the ethanol oxidation reaction was observed, thereby indicating that Pd serves as the principal active site for EOR (Figure S11, Supporting Information). The EOR activity of each material is visually demonstrated in Figure 3b and the current densities were normalized by noble metal (Pd or Pd+Rh) mass loading. The high‐entropy PdRhFeCoMo HEM demonstrated a reduced onset potential of 0.152 V compared to those of PdFeCoMo MEM (0.398 V), PdMo LEM (0.427 V), and Pd/C (0.463 V) (Figure S12, Supporting Information), indicating a diminished activation barrier for ethanol oxidation on the PdRhFeCoMo HEM surface.^[^ ^39^ ^]^ Moreover, the PdRhFeCoMo HEM showed the best EOR mass activity of 7.47 A mg_Pd+Rh_^−1^ (9.96 A mg_Pd_^−1^ when normalized by Pd, Figure S13, Supporting Information), which is 8.9, 4.6, and 1.8 times higher than those of Pd/C (0.84 A mg_Pd_^−1^), PdMo LEM (1.63 A mg_Pd_^−1^), and PdFeCoMo MEM (4.08 A mg_Pd_^−1^), respectively (Figure 3b). To elucidate the remarkable EOR mass activity and reproducibility of the PdRhFeCoMo HEM, we present both error bar data and corresponding error curves (Figure S14 and Table S3, Supporting Information). Even when the current density was normalized to the ECSAs, PdRhFeCoMo HEM still showed the best EOR‐specific activity compared to the other control samples, indicating excellent intrinsic activity (Figure 3c). Notably, the EOR activity of PdRhFeCoMo HEM also shows strong competitiveness compared to recently reported noble metal‐based catalysts, as shown in Table S2 (Supporting Information). To further investigate the stability of the as‐prepared catalysts, continuous CV cycling, and CA tests were conducted. Figure 3d and Figure S15 (Supporting Information) illustrate the mass activity of PdRhFeCoMo HEM after 1000, 2500, and 4000 cycles, showing values of 85.19%, 80.97%, and 71.04% relative to the initial activity, respectively. In contrast, the mass activity of PdFeCoMo MEM decreased to 68.89% and 41.85% of the initial value after 1000 and 2500 cycles, respectively (Figure S16a, Supporting Information). The PdMo LEM exhibited a much lower mass activity of only 19.63% and 13.49% after 1000 and 2500 cycles, respectively (Figure S16b, Supporting Information). In addition, chronoamperometry testing was also performed to assess the stability of metallene. Following 20 000 s of chronoamperometry testing, PdMo LEM displayed the lowest stability for ethanol oxidation reaction (EOR), with its mass activity dropping to 0.05 A mg_Pd_^−1^. In contrast, PdFeCoMo MEM and PdRhFeCoMo HEM maintained high EOR activities of 0.52 A mg_Pd_^−1^ and 0.56 A mg_Pd+Rh_^−1^ (0.75 A mg_Pd_^−1^), respectively (Figure 3e). Moreover, electrochemical CO stripping experiments were also conducted to evaluate the anti‐poisoning of metallenes (Figure S17, Supporting Information). The lower onset potentials indicate that the adsorbed CO_ads_ is more easily oxidized to carbon dioxide. The corresponding onset potentials are as follows: PdRhFeCoMo HEM (0.42 V) < PdFeCoMo MEM (0.57 V) < PdMo LEM (0.80 V), which is consistent with the stability results.^[^ ^40^, ^41^, ^42^, ^43^ ^]^ These results imply that the implantation of Fe and Co facilitates the removal of poisoning intermediates (CO_ads_) due to their stronger O/OH adsorption ablity, thereby enhancing EOR stabilities. To further highlight the advantages of high‐entropy metallene in terms of structural stability and mass activity, the morphology, composition, and structure of PdRhFeCoMo HEM were again examined after the stability test. XPS analysis revealed the survival of all metal elements in PdRhFeCoMo HEM even after undergoing extensive stability testing (Figure S18, Supporting Information). Importantly, the predominant zero valence state of Pd signified its sustained role as an active site in the catalytic system, further substantiating its significance in the observed EOR performance (Figure S19, Supporting Information). Transmission electron microscopy (TEM) images and the corresponding EDS elements mapping after stability testing further confirm that the PdRhFeCoMo HEM retains its initial morphology and composition (Figure S20, Supporting Information). The obtained results highlight the robust mechanical stability and notable resistance to poisoning of PdRhFeCoMo HEM, which can be attributed to the synergistic effect resulting from high entropy alloying. When comparing the five EOR performance metrics (Figure 3f), the PdRhFeCoMo HEM demonstrated superior mass activity, specific activity, and cycling stability, as well as the best *i*–*t* stability. Figure 3g presents a comparative analysis of our study with recently reported catalysts in terms of *i*–*t* stability.^[^ ^2^, ^38^, ^39^, ^41^, ^44^, ^45^, ^46^, ^47^, ^48^, ^49^ ^]^ The results unequivocally demonstrate the exceptional stability of the high‐entropy metallene PdRhFeCoMo HEM, further affirming its application potential in EOR.

**Figure 4:**
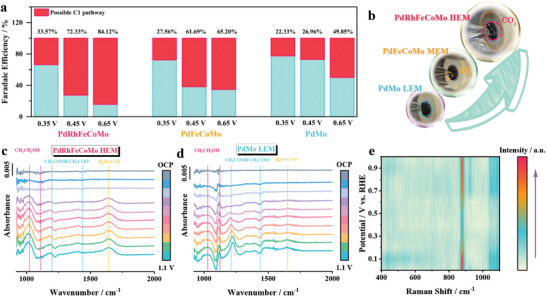
C1 selectivity of the metallenes. a) Faradaic efficiency of C2 product and possible C1 pathway. b) High‐resolution photographs during EOR. In situ FTIR spectra of c) PdRhFeCoMo HEM, and d) PdMo LEM in Ar‐saturated 1 m KOH + 1 m CH_3_CH_2_OH electrolyte. e) In situ electrochemical Raman spectroscopy maps showing the Raman signal evolution as the function of the applied anodic potential during the EOR on PdRhFeCoMo HEM in Ar‐saturated 1 M KOH + 1 M CH_3_CH_2_OH electrolyte.

Pathway selectivity is a crucial parameter in EOR reactions, providing direct insight into the fuel's energy utilization efficiency. In this study, three metallene samples (PdRhFeCoMo HEM, PdFeCoMo MEM, and PdMo LEM) and Pd/C were selected to evaluated the possible C1 pathway via the calibration curve method (Figures S21–23, Supporting Information).^[^ ^28^ ^]^ **Figure** **4**a illustrates the C1 pathway selectivity under various voltage conditions for the three metallene samples. PdRhFeCoMo HEM exhibited the highest C1 selectivity at all examined voltages. Specifically, the possible FE of PdRhFeCoMo HEM in the C1 pathway reached 84.12% at 0.65 V, significantly surpassing that of PdFeCoMo MEM (65.20%) and PdMo LEM (49.85%). In contrast, Pd/C demonstrates the lowest possible C1 selectivity of 9.6% at the same potential (Figure S24, Supporting Information). To highlight the significant advantage of PdRhFeCoMo HEM in terms of pathway selectivity, high‐resolution photographs of the three metallenes were captured during the EOR process (Figure 4b). The images reveal that PdRhFeCoMo HEM generates a considerable amount of carbon dioxide bubbles during the EOR process, confirming its exceptional C─C bond cleavage ability. In contrast, the EOR photographs of PdFeCoMo MEM shows only a small number of carbon dioxide bubbles, while no bubbles are observed for PdMo LEM. This finding provides evidence that the introduction of Rh greatly enhanced the ability to break C─C bonds. By comparing with the reported C1 pathway selectivity ratios in recent years, PdRhFeCoMo HEM exhibited the highest 12‐electron pathway selectivity ratio (Figure S25, Supporting Information). To further demonstrate the advantages of PdRhFeCoMo HEM in terms of pathway selectivity, in situ Fourier transform infrared spectroscopy (FTIR) was performed on PdMo LEM and PdRhFeCoMo HEM (Figure 4c,d). Prominent peaks at 1018 and 1113 cm^−1^ are primarily attributed to the C─O stretching vibration of ethanol, indicating ethanol consumption on the surface of PdRhFeCoMo HEM and PdMo LEM samples (Figure 4c,d).^[^ ^50^ ^]^ It is noteworthy that the two peaks appearing ≈1200–1415 cm^−1^ are attributed to the stretching vibrations of CO in acetic acid, indicating the presence of C2 pathway in both metallenes EOR processes.^[^ ^51^ ^]^ The acetic acid stretching vibration in PdMo LEM is notably more pronounced, indicating incomplete oxidation in the ethanol oxidation reaction. The peak at 1640 cm^−1^ observed in PdRhFeCoMo HEM sample, attributed to the exclusion of interfacial H_2_O by incoming CH_3_CH_2_OH, is also significantly reduced in the PdMo LEM.^[^ ^52^ ^]^ In addition, the observed 3600 cm^−1^ peak corresponds to the presence of loose OH^*^ intensity on the material surface (Figure S26a,b, Supporting Information).^[^ ^53^ ^]^ Both of the integrated OH^*^ intensity and the percentage of integrated OH^*^ intensity at 3600 cm^−1^ in interfacial water on PdRhFeCoMo HEM far surpass those on the PdMo LEM (Figure S26c,d, Supporting Information). As the voltage increases, the integrated OH^*^ on the PdRhFeCoMo HEM surface remains in dynamic equilibrium, suggesting that the introduction of Fe and Co in metallene continuously produces dangling O─H bond. The presence of O─H bond is considered to be beneficial for the oxidation and removal of adsorbed CO^*^.^[54]^ The in situ FTIR spectra provide compelling evidence that PdRhFeCoMo HEM exhibits enhanced ethanol oxidation kinetics, elevated C1 selectivity, and anti‐poisoning ability.

In situ electrochemical Raman spectroscopy (IERS) was also employed to observe the EOR process. Figure. S27 (Supporting Information) illustrates the Raman spectra acquired in 1 M KOH + 1 M CH_3_CH_2_OH from PdRhFeCoMo HEM and PdMo LEM catalysts at various potentials. The pristine electrolyte showed clear Raman signals of CH_3_CH_2_OH (Figure S28, Supporting Information), namely, bands assigned to versus(C─O─C) bonding (≈883 cm^−1^), *v_a_* (C─O─C) bonding (1043 cm^−1^) and symmetric and asymmetric vibrational *γ*(C─O─H) bonding (≈1080 cm^−1^). Since Raman signals of reaction intermediates mainly appear in the range of 400 to 1100 cm^−1^, we present a 2D IERS map showing the evolution of Raman signals as a function of applied anodic potentials, as shown in Figure 4e and Figure S29 (Supporting Information). It is noteworthy that, in comparison to PdMo LEM, the Raman signals intensity of C─O─C and C─O─H bonds on PdRhFeCoMo HEM suddenly weaken at 0.3 V and returned to original strength after 0.65 V. This variation indicates the rapid cleavage of C─C and O─H bonds on PdRhFeCoMo HEM, signifying enhanced EOR dynamics. Additionally, a Raman signal of CO₂ is observed at ≈1272 cm^−1^, demonstrating the excellent C1 selectivity of the PdRhFeCoMo HEM (Figure S26, Supporting Information). In contrast, the Raman signal of PdMo LEM exhibited minimal variation, indicating a lack of EOR activity and C1 selectivity. The in situ spectroscopy analysis above confirms that PdRhFeCoMo HEM primarily follows the C1‐12e pathway for EOR, resulting in CO_2_ and H_2_O as the final products. In contrast, PdMo LEM mainly follows the C2‐4e pathway, producing acetic acid as the primary product.

**Figure 5:**
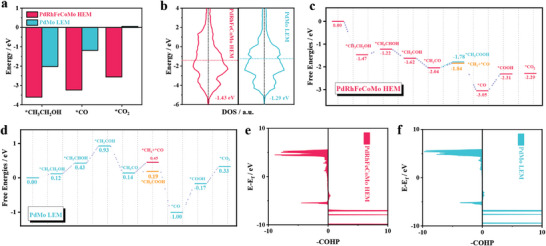
Theoretical calculation of the metallenes. a) Adsorption energy of ^*^CH_3_CH_2_OH, ^*^CO and ^*^CO_2_ on PdRhFeCoMo HEM and PdMo LEM. b) The PDOS of surface atoms over PdRhFeCoMo HEM and PdMo LEM. Free energy diagram of c) PdRhFeCoMo HEM and d) PdMo LEM. The negative integrated value of COHP (–ICOHP) of e) PdRhFeCoMo HEM and f) PdMo LEM.

Density functional theory (DFT) calculations were carried out to clarify the origin of improved EOR activities and high C1 pathway selectivity for high‐entropy PdRhFeCoMo HEM. The Pd (111) surface was selected to construct theoretical models for PdRhFeCoMo HEM and PdMo LEM configurations (Figures S30 and S31, Supporting Information). More computational details are available in Supporting Information. In terms of overall catalytic performance, the PdRhFeCoMo HEM demonstrates strong adsorption for various reaction intermediates, whereas the PdMo LEM exhibits relatively weak adsorption, especially in the initial step of the EOR process, i.e., adsorption of ethanol molecule (PdRhFeCoMo HEM: *E*_ads_ = −3.61 eV, PdMo LEM: *E*_ads_ = −2.02 eV, **Figure** **5**a), which suggests better activation of ethanol on PdRhFeCoMo HEM than PdMo LEM. The Pd atom is considered as the adsorption site of ethanol and other reaction intermediates as shown in Figures S32 and S33 (Supporting Information). To further unravel the physical mechanism underlying the interaction strength between alloys and adsorbates, the density of states (DOS) of surface layer atoms for both materials was examined (Figure 5b). The incorporation of additional elements, specifically Fe, Co, and Rh, resulted in a difference in the *d*‐band center between PdRhFeCoMo HEM and PdMo LEM. The DFT calculations revealed that the *d*‐band centers of surface layer atoms for PdMo LEM and PdRhFeCoMo HEM were −1.43 and −1.29 eV, respectively. This upward shift in the *d*‐band center contributes to the enhanced adsorption and activation of ethanol molecules on PdRhFeCoMo HEM, consistent with earlier work function measurement results.

As depicted in Figure 5c,d, simulations of the complete C1 and C2 reaction pathways for EOR were conducted on both PdRhFeCoMo HEM and PdMo LEM. The selectivity for C1/C2 routine is regarded as important performance during the EOR process which is primarily determined by the conversion tendency of the ^*^CH_3_CO intermediate. The calculations show that the PdRhFeCoMo HEM exhibits superior selectivity for the C1 pathway, while the PdMo LEM display selectivity for C2 pathway. Specifically, compared to the reaction of ^*^CH_3_CO + OH → ^*^CH_3_COOH (the C2 pathway) (*E*_barrier_ = 0.26 eV), the energy barrier for the cleavage from ^*^CH_3_CO into ^*^CH_3_ and ^*^CO is lower (*E*_barrier_ = 0.20 eV), suggesting a preference for the C1 pathway in the PdRhFeCoMo HEM. In contrast, on the PdMo LEM surface, ^*^CH_3_CO shows a low energy barrier for conversion to acetic acid (*E*_barrier_ = 0.05 eV), while the energy barrier for its cleavage into ^*^CH_3_ and ^*^CO is high (*E*_barrier_ = 0.31 eV), which makes C─C bond cleavage challenging and strongly inhibits the further formation of C1 species, thereby displaying high selectivity for the C2 pathway (Figure S31, Supporting Information). Tracing the origin of the selectivity discrepancy, the C─C bonding characteristics in ^*^CH_3_CO species were analyzed using Crystal Orbital Hamilton Population (COHP) analysis, and the integrated value of which (ICOHP) is the qualification of bond strength (Figure 5e,f). The more negative the value is, the stronger the bonding strength. For CH_3_CO adsorbed on the PdMo LEM, the C─C bond of which the bond strength (ICOHP = −3.940) is stronger than that adsorbed on the HEM (ICOHP = −3.767) revealing that on the HEM the C─C bond is notably activated than that on PdMo LEM thereby facilitating the cleavage of the C─C bond to produce C1 species, which agrees with the experimental results. Additionally, PdRhFeCoMo HEM offers sufficient CO anti‐poisoning performance than PdMo LEM which is crucial in the EOR process. The PdRhFeCoMo HEM shows a stronger adsorption energy for CO molecules (*E*_ads_ = −3.24 eV) compared to the PdMo LEM (*E*_ads_ = −1.19 eV). Nevertheless, PdRhFeCoMo HEM exhibits enhanced CO conversion rates (Δ*G*(^*^CO → ^*^COOH) = 0.74 eV) and demonstrates superior resistance to CO poisoning compared to LEM (Δ*G*(^*^CO → ^*^COOH) = 0.83 eV), which mainly owes to the enhanced adsorption of OH at Fe and Co sites in PdRhFeCoMo HEM (Figure S34, Supporting Information, see details in SI). Overall, the introduction of multiple metal elements effectively modulates the electronic properties of Pd sites, which enhances reactant adsorption and selectivity for the C1 pathway. The strong adsorption capabilities for OH further promote the oxidation of ^*^CO to CO_2_, providing excellent anti‐poisoning performance.

**Figure 6:**
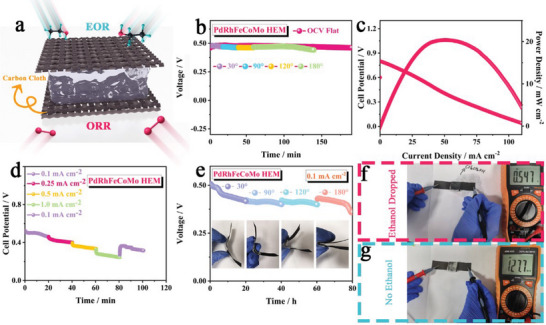
FSS‐DEFC performance of the PdRhFeCoMo HEM. a) Schematic diagram of DEFCs. b) OCV time plot under various angles. c) LSV curves and power density plots. d) Voltage‐time curves with the changes of current densities. e) Discharge curves at 0.1 mA cm^−2^ under consecutive bending from 30° to 180°. Photographs of OCV f) with enthanol, and g) without enthanol.

To further assess the potential applications of high‐entropy alloy catalysts, we assembled a flexible solid‐state direct ethanol fuel cell (FSS‐DEFC) using the prepared catalysts as the anode and our previously reported single‐atom catalyst ((CFe_2.5_)_NP_/Fe_SA_‐N‐C) as the cathode (**Figure** **6**a).^[^ ^55^ ^]^ The solid electrolyte was prepared following the procedure outlined in the literature (details in the Experimental Section).^[^ ^55^ ^]^ As depicted in Figure 6b, PdRhFeCoMo HEM FSS‐DEFC exhibits an impressive stable open‐circuit voltage of ≈0.5 V under both normal and folding conditions, surpassing the performance of a bimetallic alloy pair (Figure S35, Supporting Information). Notably, even when folded to 180 degrees, the high‐entropy alloy‐based battery maintains an open circuit voltage of 0.43 V for 80 minutes, showcasing its remarkable performance and flexibility. Figure 6c depicts the peak power density of PdRhFeCoMo HEM FSS‐DEFC is as high as 20.1 mW cm^−2^, which shows strong competitiveness than previous reports (Table S4, Supporting Information) and bimetallic alloy counterpart (0.8 mW cm^−2^, Figure S36, Supporting Information).^[^ ^47^ ^]^ Figure 6d illustrates the discharge curves of PdRhFeCoMo HEM FSS‐DEFC at various current densities. The excellent rate performance indicated that PdRhFeCoMo HEM is a high‐performance EOR catalyst that can ensure DEFC works in different application scenarios. Interestingly, the DEFC maintained a stable discharge voltage even when subjected to various bending angles, further demonstrating its outstanding flexibility in discharging and providing new possibilities for practical applications of small‐scale flexible devices (Figure 6e). In order to further evaluate the stability of FSS‐DEFC, accelerated stress tests (AST) were performed on the fuel cells assembled by the two metallenes. The power density of the FSS‐DEFC assembled with PdMo LEM is only 0.4 mW cm^−2^ after 5000 cycles, and the power density is close to zero after 10 000 cycles (Figure S37a, Supporting Information). For comparison, we also subjected fuel cells assembled with a PdRhFeCoMo HEM to the AST test. After 10 000 cycles, the power density was ≈10 mW cm^−2^, and after 20 000 cycles, it decreased to ≈5 mW cm^−2^ (Figure S37b, Supporting Information). Figure 6f,g depict the injection and operational characteristics of the connected DEFC. Without the addition of ethanol, the voltmeter registered a voltage of merely 127.7 mV. However, when a small amount of ethanol was dropped onto the anode, the open circuit voltage rapidly surged to ≈0.55 V. This provides evidence for the practical feasibility of device operation.

## 3 Conclusion

In summary, the rational design of PdRhFeCoMo HEM with carefully tailored composition and structure maximizes the utilization of active sites and the structural advantages of noble metals. CO stripping experiments and the onset potential for EOR demonstrated that the presence of oxygen affinity metals, Fe and Co, facilitated the deep oxidation of intermediate species into carbon dioxide, enhancing the stability of EOR (mass activity of 0.56 A mg_Pd+Rh_^−1^ / 0.75 A mg_Pd_^−1^ after a 20 000 s *i*–*t* test; mass activity retention of 71.04% after 4000 cycles). Combined with H^1^ NMR and in situ FTIR spectra, the introduction of Rh strengthened the C─C bond cleavage ability of the metallene, improving the selectivity toward the C1 pathway (possible C1 pathway of 84.12%). Density functional theory (DFT) calculations support that PdRhFeCoMo HEM exhibited stronger ethanol adsorption energy compared to PdMo LEM, and the synergistic effect of multimetallic components brings the *d*‐band center of Pd closer to the Fermi level. The assembled FSS‐DEFC using PdRhFeCoMo HEM demonstrates improved discharge voltage and power density (20.1 mW cm^−2^), offering new possibilities for practical applications in ethanol fuel cells.

## Conflict of Interest

The authors declare no conflict of interest.

## Supporting information



Supporting Information

## Data Availability

The data that support the findings of this study are available from the corresponding author upon reasonable request.

## References

[1] Z. Li , S. Ning , J. Xu , J. Zhu , Z. Yuan , Y. Wu , J. Chen , F. Xie , Y. Jin , N. Wang , H. Meng , S. Sun , Energy Environ. Sci. 2022, 15, 5300.

[2] F. Lv , W. Zhang , M. Sun , F. Lin , T. Wu , P. Zhou , W. Yang , P. Gao , B. Huang , S. Guo , Adv. Energy Mater. 2021, 11, 2100187.

[3] J. Chang , G. Wang , X. Chang , Z. Yang , H. Wang , B. Li , W. Zhang , L. Kovarik , Y. Du , N. Orlovskaya , B. Xu , G. Wang , Y. Yang , Nat. Commun. 2023, 14, 1346. 36906649 10.1038/s41467-023-37011-z PMC10008627

[4] J. Chang , G. Wang , C. Li , Y. He , Y. Zhu , W. Zhang , M. Sajid , A. Kara , M. Gu , Y. Yang , Joule 2023, 7, 587.

[5] L. Hui , X. Zhang , Y. Xue , X. Chen , Y. Fang , C. Xing , Y. Liu , X. Zheng , Y. Du , C. Zhang , F. He , Y. Li , J. Am. Chem. Soc. 2022, 144, 1921. 35044172 10.1021/jacs.1c12310

[6] C. Wang , Z. Huang , Y. Ding , M. Xie , M. Chi , Y. Xia , J. Am. Chem. Soc. 2022, 145, 2553. 36576951 10.1021/jacs.2c12368

[7] M. Zhao , Z. Chen , Y. Shi , Z. D. Hood , Z. Lyu , M. Xie , M. Chi , Y. Xia , J. Am. Chem. Soc. 2021, 143, 6293. 33852314 10.1021/jacs.1c02734

[8] W. Chen , S. Luo , M. Sun , X. Wu , Y. Zhou , Y. Liao , M. Tang , X. Fan , B. Huang , Z. Quan , Adv. Mater. 2022, 34, 2206276. 10.1002/adma.202206276 36063819

[9] B. Ren , H. Cui , C. Wang , Electrochem. Energy Rev. 2022, 5, 32.

[10] P. Wang , H. Cui , C. Wang , Nano Energy 2019, 66, 104196.

[11] T. Chen , S. Xu , T. Zhao , X. Zhou , J. Hu , X. Xu , C. Liang , M. Liu , W. Ding , Angew. Chem., Int. Ed. 2023, 62, e202308057. 10.1002/anie.202308057 37545437

[12] Y. Qiu , J. Zhang , J. Jin , J. Sun , H. Tang , Q. Chen , Z. Zhang , W. Sun , G. Meng , Q. Xu , Y. Zhu , A. Han , L. Gu , D. Wang , Y. Li , Nat. Commun. 2021, 12, 5273. 34489455 10.1038/s41467-021-25600-9 PMC8421426

[13] K. Wei , H. Lin , X. Zhao , Z. Zhao , N. Marinkovic , M. Morales , Z. Huang , L. Perlmutter , H. Guan , C. Harris , M. Chi , G. Lu , K. Sasaki , S. Sun , J. Am. Chem. Soc. 2023, 145, 19076. 37606196 10.1021/jacs.3c07027

[14] S. Luo , L. Zhang , Y. Liao , L. Li , Q. Yang , X. Wu , X. Wu , D. He , C. He , W. Chen , Q. Wu , M. Li , E. J. M. Hensen , Z. Quan , Adv. Mater. 2021, 33, 2008508. 10.1002/adma.202008508 33749954

[15] J. Hao , Z. Zhuang , K. Cao , G. Gao , C. Wang , F. Lai , S. Lu , P. Ma , W. Dong , T. Liu , M. Du , H. Zhu , Nat. Commun. 2022, 13, 2662. 35562523 10.1038/s41467-022-30379-4 PMC9106752

[16] L. Hu , Y. Zhang , H. Wu , J. Li , Y. Li , M. McKenna , J. He , F. Liu , S.‐J. Pennycook , X. Zeng , Adv. Energy Mater. 2018, 8, 1802116.

[17] B. Jiang , Y. Yu , J. Cui , X. Liu , L. Xie , J. Liao , Q. Zhang , Y. Huang , S. Ning , B. Jia , B. Zhu , S. Bai , L. Chen , S.‐J. Pennycook , J. He , Science 2021, 371, 830. 33602853 10.1126/science.abe1292

[18] X. Lao , X. Liao , C. Chen , J. Wang , L. Yang , Z. Li , J.‐W. Ma , A. Fu , H. Gao , P. Guo , Angew. Chem., Int. Ed. 2023, 62, e202304510. 10.1002/anie.202304510 37278913

[19] H. Li , Y. Pan , D. Zhang , Y. Han , Z. Wang , Y. Qin , S. Lin , X. Wu , H. Zhao , J. Lai , B. Huang , L. Wang , J. Mater. Chem. A. 2020, 8, 2323.

[20] S. Zhang , Z. Zeng , Q. Li , B. Huang , X. Zhang , Y. Du , C.‐H. Yan , Energy Environ. Sci. 2021, 14, 5911.

[21] F. Lin , F. Lv , Q. Zhang , H. Luo , K. Wang , J. Zhou , W. Zhang , W. Zhang , D. Wang , L. Gu , S. Guo , Adv. Mater. 2022, 34, 2202084. 10.1002/adma.202202084 35484940

[22] M. Luo , Z. Zhao , Y. Zhang , Y. Sun , Y. Xing , F. Lv , Y. Yang , X. Zhang , S. Hwang , Y. Qin , J.‐Y. Ma , F. Lin , D. Su , G. Lu , S. Guo , Nature 2019, 574, 81. 31554968 10.1038/s41586-019-1603-7

[23] J. Zhang , F. Lv , Z. Li , G. Jiang , M. Tan , M. Yuan , Q. Zhang , Y. Cao , H. Zheng , L. Zhang , C. Tang , W. Fu , C. Liu , K. Liu , L. Gu , J. Jiang , G. Zhang , S. Guo , Adv. Mater. 2022, 34, 2105276. 10.1002/adma.202105276 34738668

[24] J. Liu , Q. Wang , T. Li , Y. Wang , H. Li , A. Cabot , Nano Res. 2023, 16, 2041.

[25] J. Fan , Z. Feng , Y. Mu , X. Ge , D. Wang , L. Zhang , X. Zhao , W. Zhang , D.‐J. Singh , J. Ma , L. Zheng , W. Zheng , X. Cui , J. Am. Chem. Soc. 2023, 145, 5710. 36877096 10.1021/jacs.2c11692

[26] X. Li , P. Shen , Y. Luo , Y. Li , Y. Guo , H. Zhang , K. Chu , Angew. Chem., Int. Ed. 2022, 61, e202205923. 10.1002/anie.202205923 35522475

[27] H. Yu , T. Zhou , Z. Wang , Y. Xu , X. Li , L. Wang , H. Wang , Angew. Chem., Int. Ed. 2021, 60, 12027. 10.1002/anie.202101019 33559316

[28] Y. Qin , H. Huang , W. Yu , H. Zhang , Z. Li , Z. Wang , J. Lai , L. Wang , S. Feng , Adv. Sci. 2022, 9, 2103722. 10.1002/advs.202103722 PMC8844492 34951154

[29] S.‐H. Han , H.‐M. Liu , P. Chen , J.‐X. Jiang , Y. Chen , Adv. Energy Mater. 2018, 8, 1801326.

[30] F. Lv , B. Huang , J. Feng , W. Zhang , K. Wang , N. Li , J. Zhou , P. Zhou , W. Yang , Y. Du , D. Su , S. Guo , Nat. Sci. Rev. 2021, 8, nwab019. 10.1093/nsr/nwab019 PMC8433090 34691734

[31] L. Chen , L. Lu , H. Zhu , Y. Chen , Y. Huang , Y. Li , L. Wang , Nat. Commun. 2017, 8, 14136. 28071650 10.1038/ncomms14136 PMC5234093

[32] J. Chang , G. Wang , M. Wang , Q. Wang , B. Li , H. Zhou , Y. Zhu , W. Zhang , M. Omer , N. Orlovskaya , Q. Ma , M. Gu , Z. Feng , G. Wang , Y. Yang , Nat. Energy 2021, 6, 1144.

[33] G. Liu , W. Zhou , Y. Ji , B. Chen , G. Fu , Q. Yun , S. Chen , Y. Lin , P.‐F. Yin , X. Cui , J. Liu , F. Meng , Q. Zhang , L. Song , L. Gu , H. Zhang , J. Am. Chem. Soc. 2021, 143, 11262. 34281338 10.1021/jacs.1c05856

[34] Z. Jin , J. Lv , H. Jia , W. Liu , H. Li , Z. Chen , X. Lin , G. Xie , X. Liu , S. Sun , H.‐J. Qiu , Small 2019, 15, 1904180. 10.1002/smll.201904180 31596058

[35] Y. Zhou , W. Hao , X. Zhao , J. Zhou , H. Yu , B. Lin , Z. Liu , S.‐J. Pennycook , S. Li , H.‐J. Fan , Adv. Mater. 2022, 34, 2100537. 10.1002/adma.202100537 34951727

[36] C. Chen , X.‐T. Wang , J.‐H. Zhong , J. Liu , G. I. N. Waterhouse , Z.‐Q. Liu , Angew. Chem., Int. Ed. 2021, 60, 22043. 10.1002/anie.202109207 34374478

[37] B. Xu , T. Liu , X. Liang , W. Dou , H. Geng , Z. Yu , Y. Li , Y. Zhang , Q. Shao , J. Fan , X. Huang , Adv. Mater. 2022, 34, 2206528. 10.1002/adma.202206528 36120846

[38] P.‐F. Yin , M. Zhou , J. Chen , C. Tan , G. Liu , Q. Ma , Q. Yun , X. Zhang , H. Cheng , Q. Lu , B. Chen , Y. Chen , Z. Zhang , J. Huang , D. Hu , J. Wang , Q. Liu , Z. Luo , Z. Liu , Y. Ge , X.‐J. Wu , X.‐W. Du , H. Zhang , Adv. Mater. 2020, 32, 2000482. 10.1002/adma.202000482 32253801

[39] H. Lv , L. Sun , Y. Wang , S. Liu , B. Liu , Adv. Mater. 2022, 34, 2203612. 10.1002/adma.202203612 35640570

[40] S. Bai , Y. Xu , K. Cao , X. Huang , Adv. Mater. 2021, 33, 2005767. 10.1002/adma.202005767 33314444

[41] H. Lv , L. Sun , D. Xu , B. Liu , Sci. Bull. 2020, 65, 1823. 10.1016/j.scib.2020.05.005 36659122

[42] H. Li , Y. Han , H. Zhao , W. Qi , D. Zhang , Y. Yu , W. Cai , S. Li , J. Lai , B. Huang , L. Wang , Nat. Commun. 2020, 11, 5437. 33116124 10.1038/s41467-020-19277-9 PMC7595151

[43] P. Wang , H. Cui , C. Wang , Chem. Eng. J. 2022, 429, 132435.

[44] W. Huang , X. Kang , C. Xu , J. Zhou , J. Deng , Y. Li , S. Cheng , Adv. Mater. 2018, 30, 1706962. 10.1002/adma.201706962 29337397

[45] T. Jiang , J. Gao , Z. Jin , K. Hu , Q. Yuan , H.‐J. Qiu , Mater. Today Energy 2021, 21, 100835.

[46] S. Li , J. Shu , S. Ma , H. Yang , J. Jin , X. Zhang , R. Jin , Appl. Catal. B Environ. 2021, 280, 119464.

[47] S. Li , J. Wang , X. Lin , G. Xie , Y. Huang , X. Liu , H.‐J. Qiu , Adv. Funct. Mater. 2021, 31, 2007129.

[48] H. Lv , X. Guo , L. Sun , D. Xu , B. Liu , Sci.China Chem. 2021, 64, 245.

[49] J. Zhang , J. Ye , Q. Fan , Y. Jiang , Y. Zhu , H. Li , Z. Cao , Q. Kuang , J. Cheng , J. Zheng , Z. Xie , J. Am. Chem. Soc. 2018, 140, 11232. 30117323 10.1021/jacs.8b03080

[50] Y. Zhang , X. Liu , T. Liu , X. Ma , Y. Feng , B. Xu , W. Cai , Y. Li , D. Su , Q. Shao , X. Huang , Adv. Mater. 2022, 34, 2202333. 10.1002/adma.202202333 35676861

[51] W. Yan , G. Li , S. Cui , G.‐S. Park , R. Oh , W. Chen , X. Cheng , J.‐M. Zhang , W. Li , L.‐F. Ji , O. Akdim , X. Huang , H. Lin , J. Yang , Y.‐X. Jiang , S.‐G. Sun , J. Am. Chem. Soc. 2023, 145, 17220. 37492900 10.1021/jacs.3c04320

[52] H. Wang , H.‐D. Abruña , J. Am. Chem. Soc. 2023, 145, 6330. 36898001 10.1021/jacs.2c13401

[53] N. Ye , P. Zhao , X. Qi , W. Sheng , Z. Jiang , T. Fang , Appl. Catal. B Environ. 2022, 314, 121473.

[54] X. Zhou , Y. Ma , Y. Ge , S. Zhu , Y. Cui , B. Chen , L. Liao , Q. Yun , Z. He , H. Long , L. Li , B. Huang , Q. Luo , L. Zhai , X. Wang , L. Bai , G. Wang , Z. Guan , Y. Chen , C.‐S. Lee , J. Wang , C. Ling , M. Shao , Z. Fan , H. Zhang , J. Am. Chem. Soc. 2022, 144, 547. 34932339 10.1021/jacs.1c11313

[55] Y. Guo , C. Wang , Y. Xiao , X. Tan , J. Chen , W. He , Y. Li , H. Cui , C. Wang , Nano Energy 2023, 117, 108895.

