# Supplementary material for: High‐Entropy PdRhFeCoMo Metallene With High C1 Selectivity and Anti‐Poisoning Ability for Ethanol Electrooxidation
Source: Adv Sci (Weinh). 2024 Nov 19;11(48):2409109. doi: 10.1002/advs.202409109 (PMC11672265; doi:10.1002/advs.202409109)
Supplement: Supplementary file 1 — Supporting Information [file ADVS-11-2409109-s001.docx]

***Supporting Information***

**High-entropy PdRhFeCoMo Metallene with High C1 selectivity and Anti-poisoning Ability for Ethanol Electrooxidation**

*Xiaohong Tan, Chenhui Wang, Jiarui Wang, Peng Wang, Yuhang Xiao, Yingying Guo, Jianpo Chen, Weidong He, Yan Li, Hao Cui,* Chengxin Wang**

School of Materials Science and Engineering, Sun Yat-sen University, Guangzhou 510275, China.

E-mail: cuihao3@mail.sysu.edu.cn (H. Cui), wchengx@mail.sysu.edu.cn (C. Wang)

**Keywords:** High-entropy metallene • Ethanol oxidation reaction • Elevated C1 selectivity • Anti-poisoning ability • Flexible solid-state fuel cell

Table of Contents

1. Experimental Section
2. Computational Section
3. Supplementary Figures and Tables
4. References
5. **Experimental Section**

**Chemicals:** Acetylacetone palladium (Pd(acac)_2_, 99%) and hexacarbonyl molybdenum (Mo(CO)_6_, 99.99%) were purchased from Sigma-Aldrich. Rhodium acetylacetonate (Rh(acac)_3_, 99.99%), iron acetylacetonate (Fe(acac)_3_, 98%), cobalt acetylacetonate (Co(acac)_2_, 98%), oleic amine (OAM, C18, 80%-90%), and potassium hydroxide (KOH, 85%) were purchased from Macklin. Commercial palladium carbon (Tianjin Biochemart Chemical Technology Co., Ltd.) and all chemicals were used as received without further purification. Ultra-pure water (18.2 MΩ‧cm) was used for all experiments. PEG-PPG-PEG Pluronic P123 (Mw = 5800).

**Synthesis of PdRhFeCoMo HEM:** By employing a conventional synthesis protocol, 5 mg of palladium acetylacetonate, 6 mg of rhodium acetylacetonate, 16 mg of iron acetylacetonate, 15 mg of cobalt acetylacetonate, and 15 mg of molybdenum acetylacetonate were introduced into a high-pressure resistant glass tube. The solid materials were dissolved in 10 mL of oleylamine and subjected to an oil bath heating at 180 °C for 8 hours. Subsequently, the resulting mixture underwent repetitive centrifugal washing with ethanol and cyclohexane, followed by a freeze-drying process lasting 12 hours, ultimately yielding the desired high-entropy metallene (PdRhFeCoMo HEM).

**Synthesis of PdFeCoMo MEM:** By employing a similar approach, 5 mg of palladium acetylacetonate, 16 mg of iron acetylacetonate, 15 mg of cobalt acetylacetonate, and 15 mg of molybdenum acetylacetonate were introduced into a high-pressure resistant glass tube. The solid materials were dissolved in 10 mL of oleylamine and heated at 180°C for 8 hours using an oil bath. After centrifugal washing with ethanol and cyclohexane, followed by freeze-drying for 12 hours, the resulting product was obtained as the desired medium-entropy metallene (PdFeCoMo MEM).

**Synthesis of PdMo LEM:** By employing a conventional synthesis method, 4 mg of palladium acetylacetonate and 10 mg of molybdenum acetylacetonate were added to a high-pressure resistant glass tube. The solid materials were dissolved in 10 mL of oleylamine and heated at 180°C for 8 hours in an oil bath. Following repetitive centrifugal washing with ethanol and cyclohexane, the resulting product was freeze-dried for 12 hours, yielding the desired bimetallic metalene (PdMo LEM).

**Characterizations:** The X-ray diffraction (XRD) patterns were acquired using the Rigaku X-ray diffractometer D-MAX 2200 VPC, employing Cu Kα radiation (λ = 0.15418 nm). The scanning step size utilized was 3 (° min^-1^). Transmission electron microscopy (TEM), high-resolution TEM (HRTEM) and high angle annular dark field scanning transmission electron microscopy (HAADF) images were taken on an FEI Tecnai G2 F30 microscope operated at 300 kV. X-ray photoelectron spectroscopy (XPS) measurements were performed on an ESCA Lab250 spectrometer using a twin-anode Al Kα (1486.6 eV) X-ray source. The relative composition of each catalyst was determined using an inductively coupled plasma atomic emission spectrometer (ICP-AES, PerkinElmer Optima 8300). Acetic acid detection was performed using a nuclear magnetic resonance spectrometer (Bruker Avance III, 400 MHz). In-situ electrochemical infrared spectroscopy (Thermo iS50 FT-IR, 32 scans) was used to detect intermediate species.

**Electrode preparation and electrocatalytic measurement:** The Autolab workstation was used to collect electrochemical data in the three-electrode system. The working electrode is a glassy carbon (GC) electrode with catalyst, and a graphite rod and an Ag/AgCl electrode are used as the counter electrode and the reference electrode, respectively. The potential, measured against an Ag/AgCl electrode, was converted to the potential versus the reversible hydrogen electrode (RHE). E_RHE_=E_Ag/AgCl_ + 0.059 pH + 0.197. Then prepare the working electrode, weigh 1 mg of powdered sample catalyst, and dissolve it into a mixture solution (100 μL of 0.5 wt% Nafion aqueous solution, 700 μL of ethanol and 200 μL of deionized water), and then ultrasonic treatment for 30 min to obtain uniformly dispersed catalyst slurry. Then, 5 μL of this catalyst ink was pipetted onto the glassy carbon (GC) electrode (0.19625 cm^2^), and dried at room temperature. The working electrodes were first activated by cycling at 500 mV s^-1^ in 1 M KOH. For the EOR test, the working electrodes were conducted at CV scans between 0.0 and 1.1 V at 50 mV s^-1^ in 1 M KOH + 1M ethanol. The mass activity and specific activity were normalized by the mass loading of noble metals (determined by ICP-AES) and electrochemically active surface area (ECSA) of catalysts. The chronoamperometry measurements were conducted at 0.65 V in the same solution. The CO stripping test were carried out in 1 M KOH solution.

**Flexible solid-state direct ethanol fuel cell (FSS-DEFC) assembly:** 1.368 g of ammonium persulphate was dissolved in 20 mL of ultrapure water with violent stirring (marked as solution A). 60 mg of ZnO, 1.89 g of potassium hydroxide and 75 mg of N, N′-methylene-bisacrylamide were dissolved in 3 mL of ultrapure water (marked as solution B). Solution B was quickly poured into a mould and then 30 microlitres of solution A was evenly distributed in the mould. After cooling, an electrolyte gel film (2 mm thick) was obtained.

**Calculation of C1 selectivity:** As a preliminary step, a series of acetic acid standard solutions have been prepared with graded concentrations (0.4-2 ppm). The standard curve can be plotted using the integral area of the acetate peak and the concentration of the standard solution (^1^H NMR). Next, the electrolyte was subjected to a long-term i-t test to collect the product. The concentration of acetic acid produced can be calculated from the standard curve. The Faradaic efficiency (FE) of EOR to CO_2_ or acetate was calculated as follows:

FE = (N × n× 96485)/Q ×100%

N is the moles of acetic acid, n is the number of electron transfers, Q is the total amount of charge consumed during the i-t test. The total FE of EOR was assumed to be 100%, the possible C1 selectivity is the remainder of the C2 pathway.

1. **Computational Section**

The first-principles calculations in the current study were performed by using the Vienna Ab initio Simulation Package (VASP) ^[1,2]^. The Perdew-Burke-Ernzerhof functional ^[3,4]^ was employed for calculating the exchange-correlation energy within the generalized gradient approximation^[5]^ and the projector augmented wave method^[6,7]^ to describe the electron-ion interactions. In addition, DFT-D3^[8]^ approach was employed to take van der Waals (vdW) interactions into account. Plane-wave cutoff energy was set at 520 eV in all the calculations. The structures of HEM and LEM were entirely optimized with the force and energy threshold of 0.02 eV Å^-1^ and 10^-4^ eV, respectively. K-point mesh for the structural optimizations (OPT), self-consistent field (SCF) calculations and non-self-consistent field (NSCF) calculation is 1×1×1, 3×3×1 and 5×5×1, respectively.

The adsorption energy (*E_ads_*) of the molecule (CH_3_CH_2_OH, CO and CH_3_COOH) on the substrate is calculated by:

where *E*_total_ is the total energy of the substrate with absorbed molecule or atom, *E*_sub_ is the total energy of the substrate, and *E*_adsorbate_ is the energy of adsorbate.

The Gibbs free energy (*G*) was obtained using the computational hydrogen electrode (CHE) ^[11]^ model. At the standard hydrogen electrode (SHE), the chemical potential of H^+^/e^−^ pair in aqueous solution is equal to half that of hydrogen gas. According to the CHE model, the free energy change of reaction (Δ*G*) for each elementary step of EOR is expressed as

where Δ*E* is the change in total energies of the species involved in each step, Δ*S* and Δ*ZPE* are the change in entropy and zero−point energies, respectively. The entropies of the absorbed intermediates were set to zero. The zero-point energies of both gas molecules and intermediates were calculated using VASP and could be determined as *ZPE* = ½Σ*_i_hν_i_*, here *h* and *ν_i_* are Planck constant and vibrational frequencies, respectively. *T* is the temperature and was set as 298.15 K here.

The *d* band center, *ε_d_*, was calculated as：

where *ρ* and *ε* refer to the projected density of states (PDOS) of *d* orbital and energy level, respectively.

We explored the C1/C2 pathway of EOR in an alkaline solution whose reactions are denoted as follows, in which * refers to the adsorption site. The process of EOR:

where **Eq.5-8** are for the common process of C1 and C2 pathway, **Eq.9** show the final reaction of C2 pathway and **Eq.10-13** describe the rest reactions of C1 pathways. The energies of various adsorbates are placed in **TableS5**.

LEM and HEM was built by introducing Mo and Fe/Co/Rh/Mo into a slab model of Pd (111), respectively, containing four metal layers and the concentrations of heteroatoms were determined by the experiments as mentioned before, as shown in (Figure S31). The PdMo LEM model consists of 72 Pd atoms and 8 Mo atoms within the simulation box with *a* = 11.09 Å, *b* = 13.87 Å, *c* = 21.74 Å, *α* = *β* = 90°, *γ* = 120°, while the FePdCoRhMo HEM is modeled by the same simulation box containing 16/24/12/20/8 atoms, respectively. The distributions of those heteroatoms in Pd (111) slab were determined by using the Monte Carlo Special Quasirandom Structures (MCSQS) module from the Alloy Theoretic Automated Toolkit (ATAT) ^[9-10]^. The MCSQS method is particularly effective for constructing supercells that accurately reflect the statistical properties of a truly random alloy, ensuring precise modeling of atomic distributions in multicomponent systems. It is well known that one cannot visit all of the possible structures of HEM/LEM and therefore we just examined 3 models (See Figure S31) and the most energetically favorable configuration (Figure S30.a2, S30.b2, S30.a3 and S30.b3) was selected for exploring the catalytic performance. During the structural relaxation, the bottom layer was fixed and 0.8% tensile strain was applied to match the physical conditions of the experimental sample. We include the structural information in the supplementary “Data File” of those two models of HEM and LEM with atomic positions in the format of POSCAR for VASP.

**For the CO anti-poisoning performance**: The conversion reaction of *CO was further investigated. As shown in Figures S32a and b, the adsorption properties of OH on different elements of the catalysts were assessed. For the LEM, Pd sites exhibit poor OH adsorption (*E*_ads_ = 0.51 eV), while Mo sites show relatively effective adsorption (*E*_ads_ = -0.89 eV), facilitating the conversion of *CO to *COOH and providing certain efficiency in *CO conversion (Δ*G*(*CO → *COOH) = 0.83 eV). Conversely, due to the existence of Fe, Co, and Rh, the HEM exhibits a significantly enhanced effect for the conversion of *CO to *COOH (Δ*G*(*CO → *COOH) = 0.74 eV), primarily due to the strong OH adsorption at Fe (*E*_ads_ = -2.74 eV) and Co (*E*_ads_ = -2.80 eV) sites. This robust adsorption effect supplies enough hydroxyl for *CO conversion, thereby enhancing the overall CO anti-poisoning performance, which is consistence with the results of experiment.

1. **Supplementary Figures and Tables**

**Figure S1**. TEM image of PdRhFeCo catalysts.

**
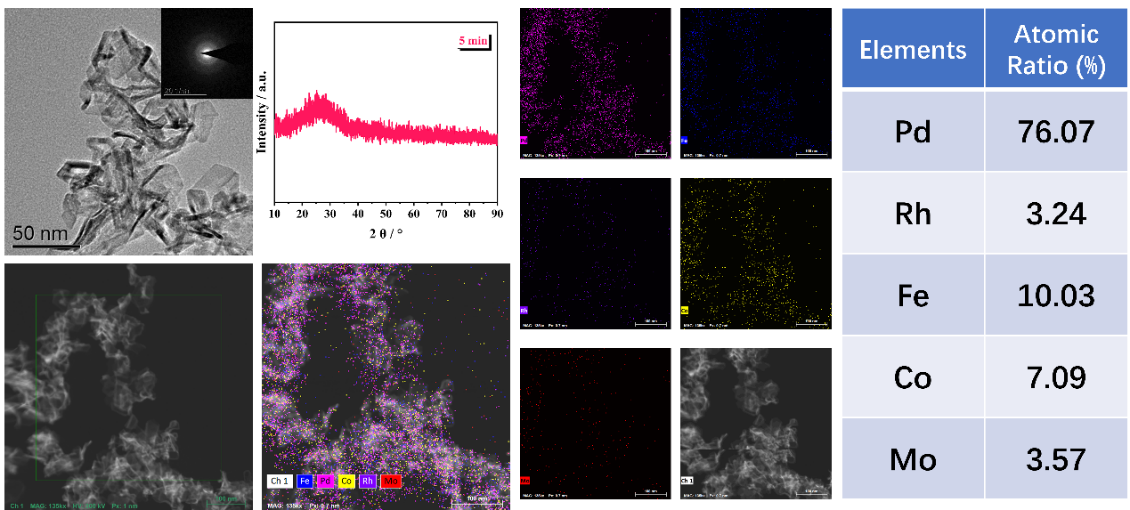
Figure S2.** TEM images, EDS spectra and XRD patterns of PdRhFeCoMo HEM collected at a reaction time of 5 minutes.


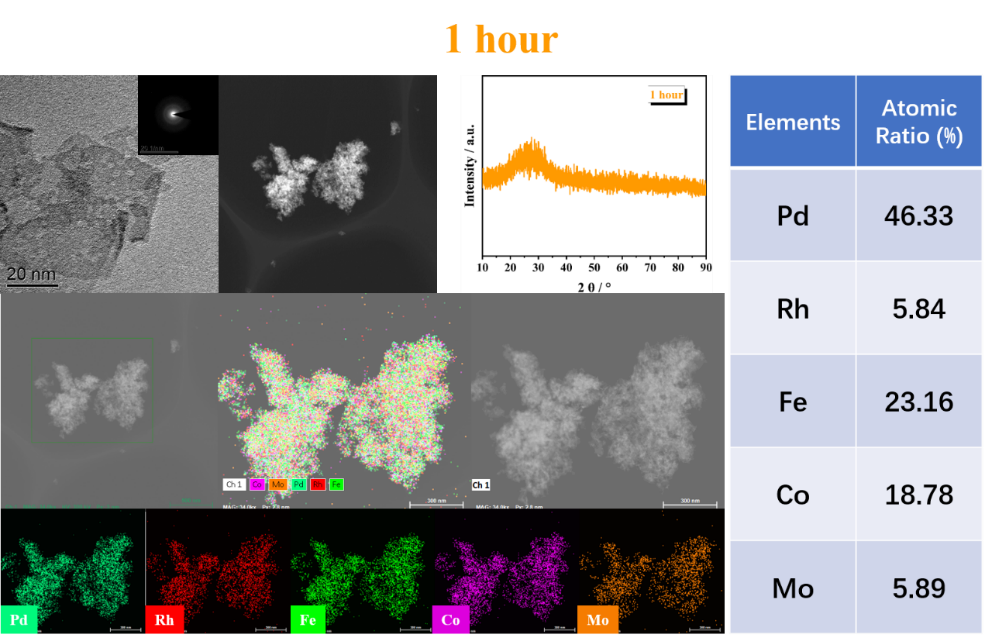


**Figure S3.** TEM images, EDS spectra and XRD patterns of PdRhFeCoMo HEM collected at a reaction time of 1 hour.


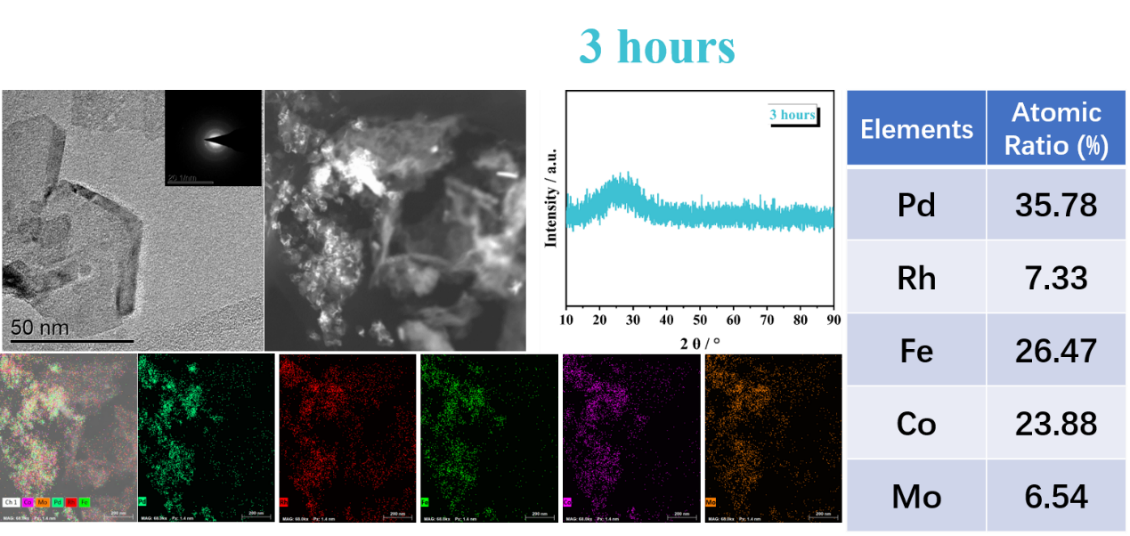


**Figure S4.** TEM images, EDS spectra and XRD patterns of PdRhFeCoMo HEM collected at a reaction time of 3 hours.



**Figure S5**. TEM image of RhFeCoMo.


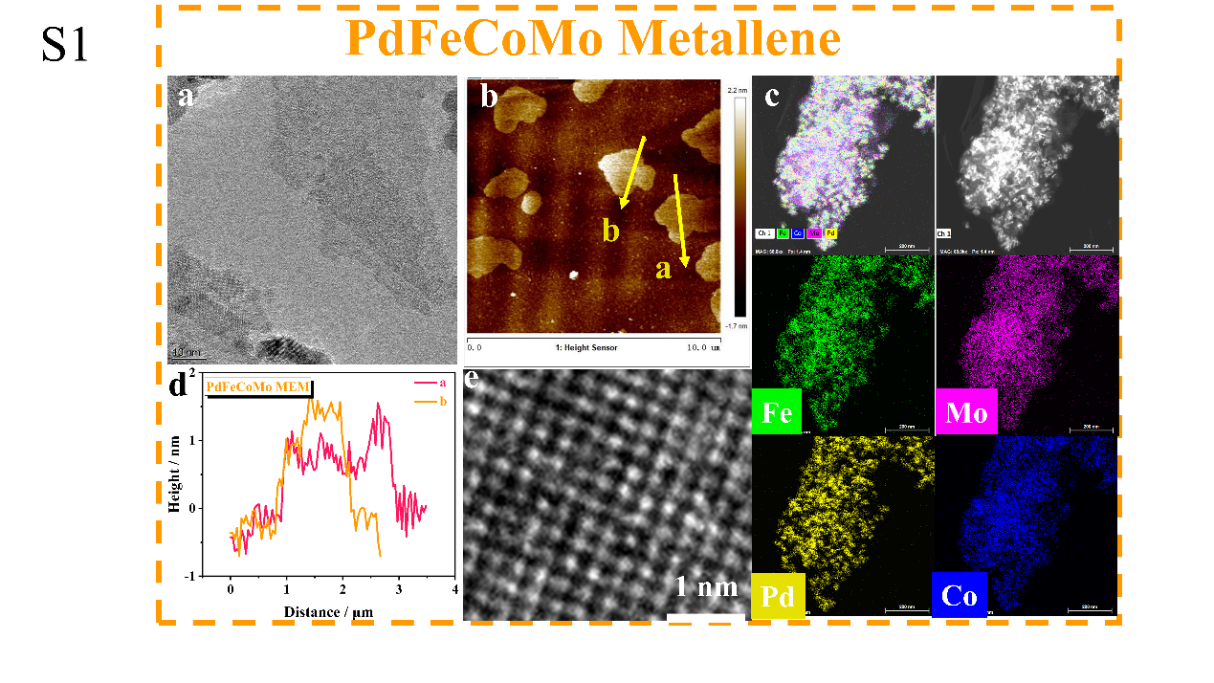


**Figure S6**. a) TEM images of PdFeCoMo MEM. b) AFM image of PdFeCoMo MEM c) EDS mapping. d) Height profile across PdFeCoMo MEM. e) HRTEM image.


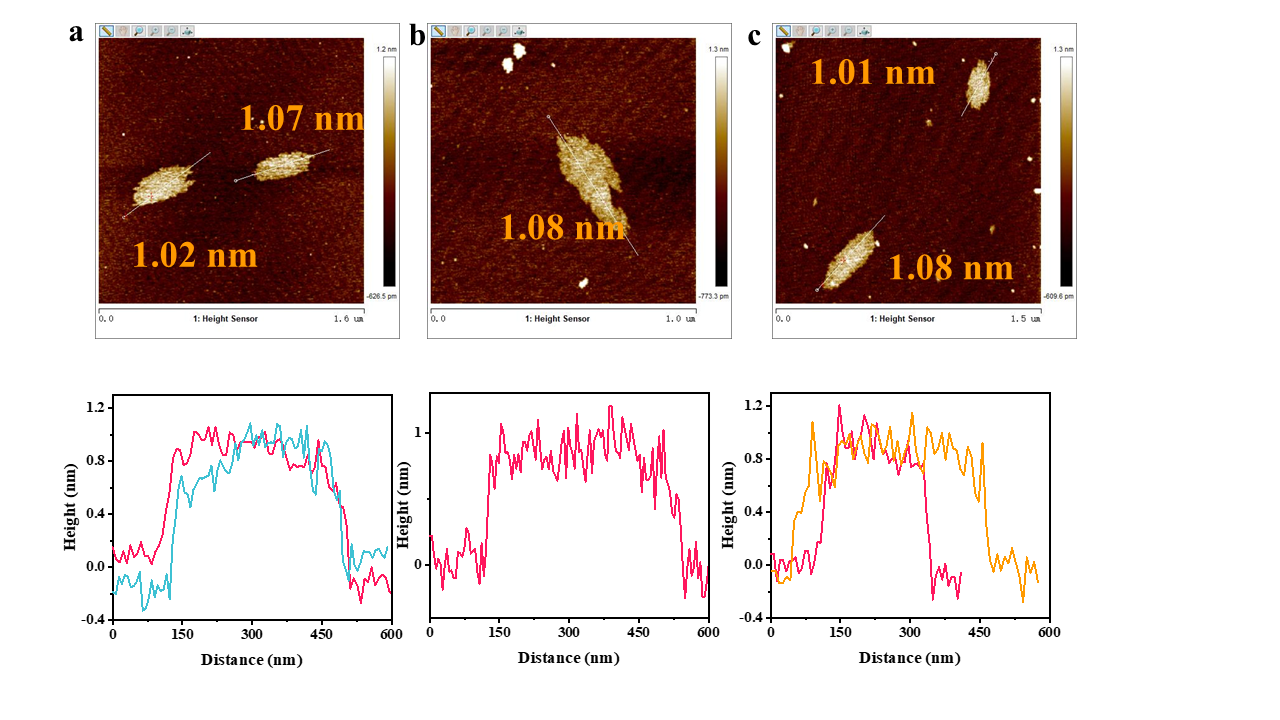
**
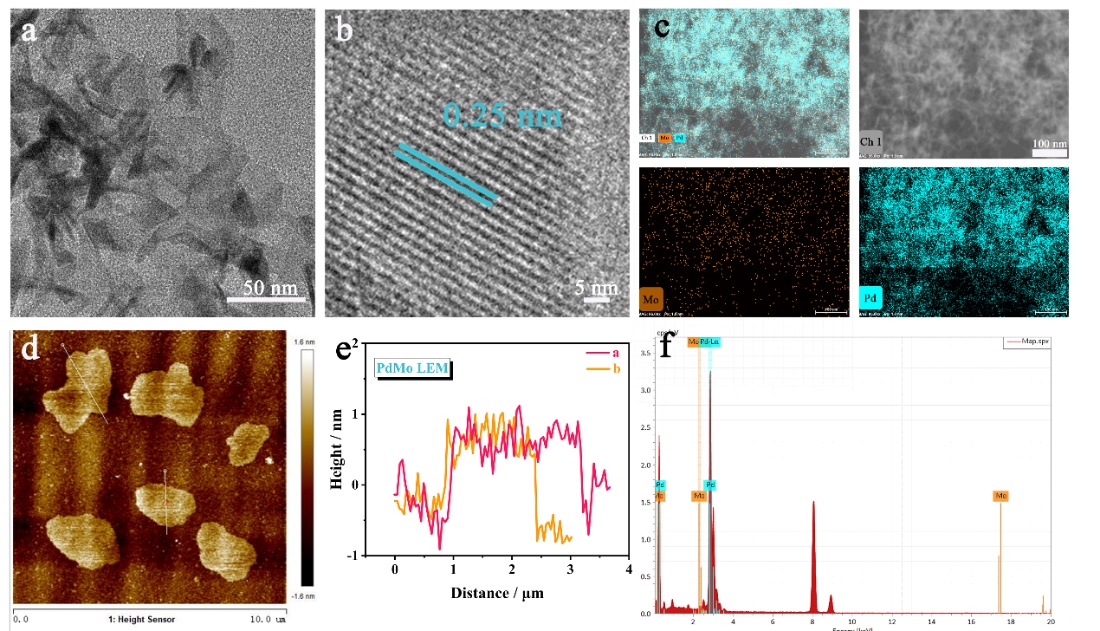
Figure S7**. a) TEM images of PdMo LEM and b) HRTEM image. c) EDS mapping of PdMo LEM, d) AFM image and e) corresponding height profile across PdMo LEM. f) EDS spectrum.

**Figure S8.** a-c) AFM images of three PdRhFeCoMo HEM samples and its corresponding height profile across a high-entropy metallene.

**
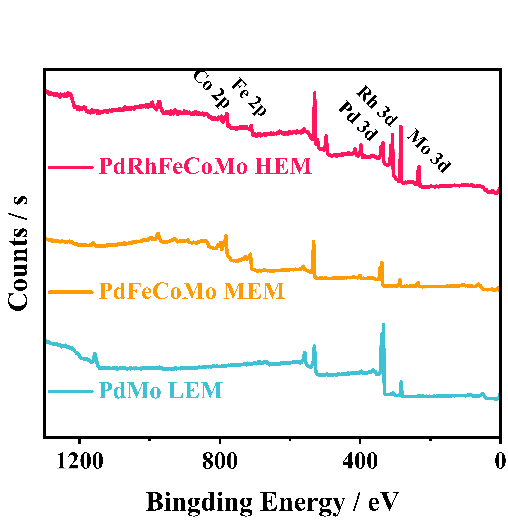
**

**Figure S9**. X-ray photoelectron spectroscopy of survey.

**Figure S10**. X-ray photoelectron spectroscopy of a) Rh 3d, b) Fe 2p, c) Co 2p, d) Mo 3d.

**Figure S11.** EOR current of RhFeCoMo and FeCoMo.


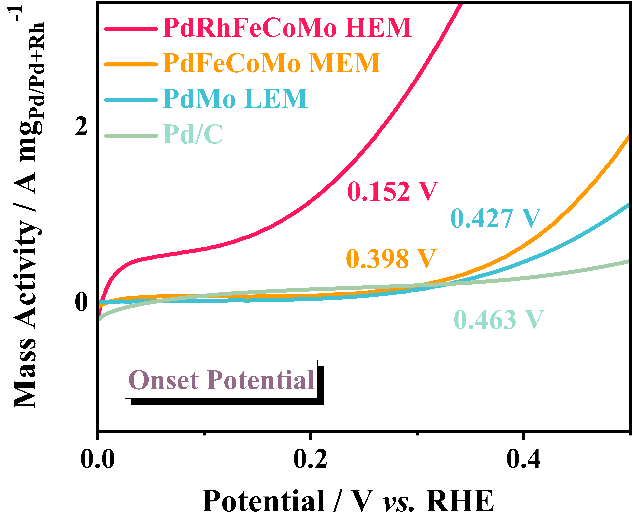


**Figure S12.** Onset potential of all samples in EOR.

**Figure S13.** Mass activities of all samples based on Pd loading.

**Figure S14.** EOR activity of PdRhFeCoMo HEM. a) Error curve of CVs. b) Error bar of mass activity.

**Figure S15.** Mass activities of PdRhFeCoMo HEM after different CV cycles.

**
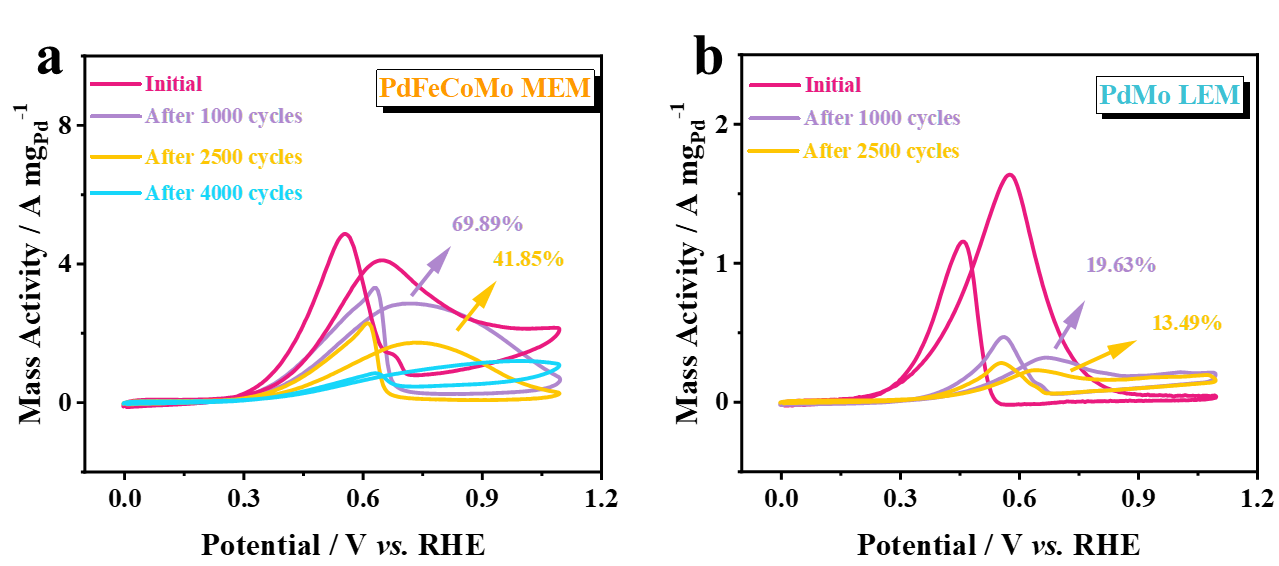
**

**Figure S16.** Mass activities of a) PdFeCoMo MEM and b) PdMo LEM after different CV cycles.

**Figure S17.** CO-stripping curves of PdRhFeCoMo HEM, PdFeCoMo MEM and PdMo LEM in Ar-saturated 1 M KOH at a scan rate of 50 mV s^-1^.


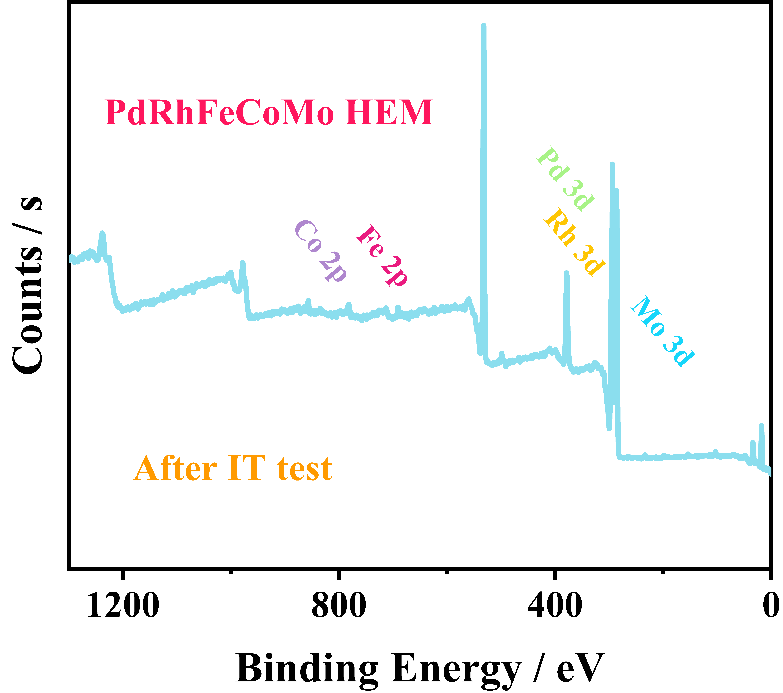


**Figure S18**. X-ray photoelectron spectroscopy of survey after IT test.

**
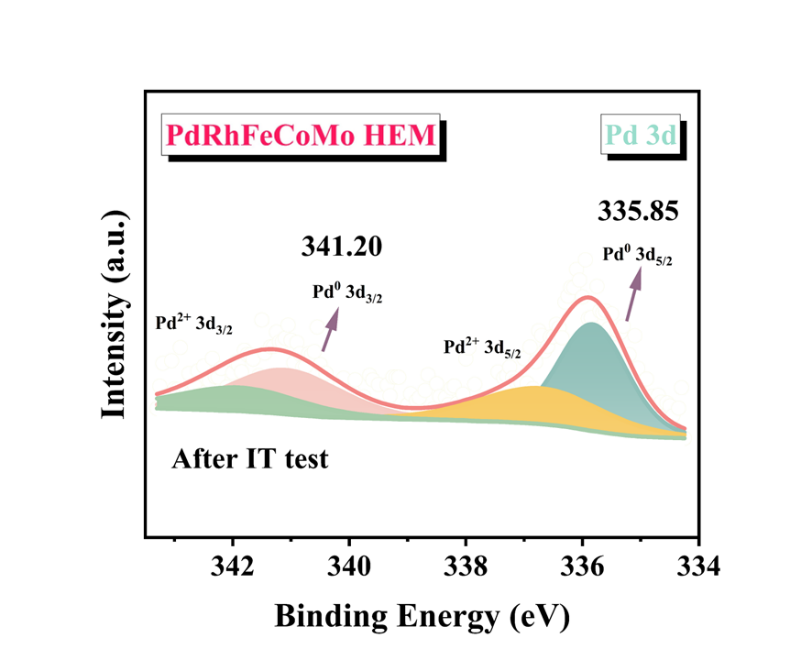
Figure S19**. X-ray photoelectron spectroscopy of PdRhFeCoMo HEM after long-term chronoamperometry for EOR at 0.65 V versus RHE.


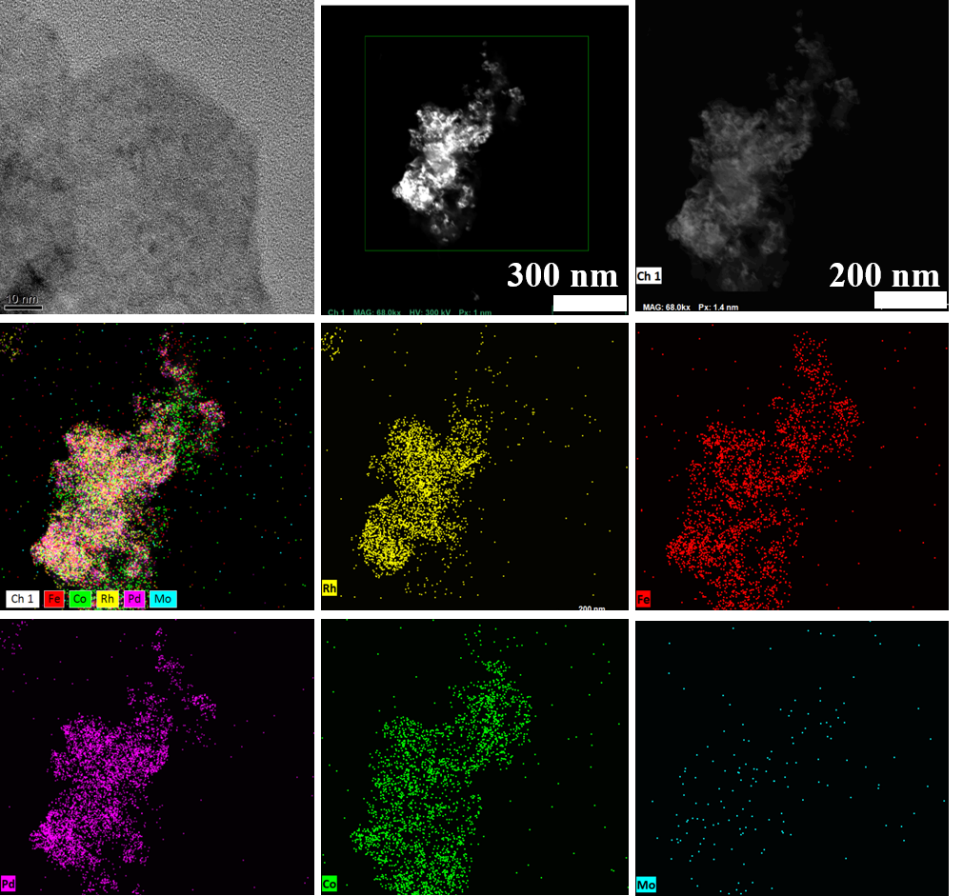


**Figure S20**. TEM images of PdRhFeCoMo HEM and its corresponding element distribution after long-term chronoamperometry.

**
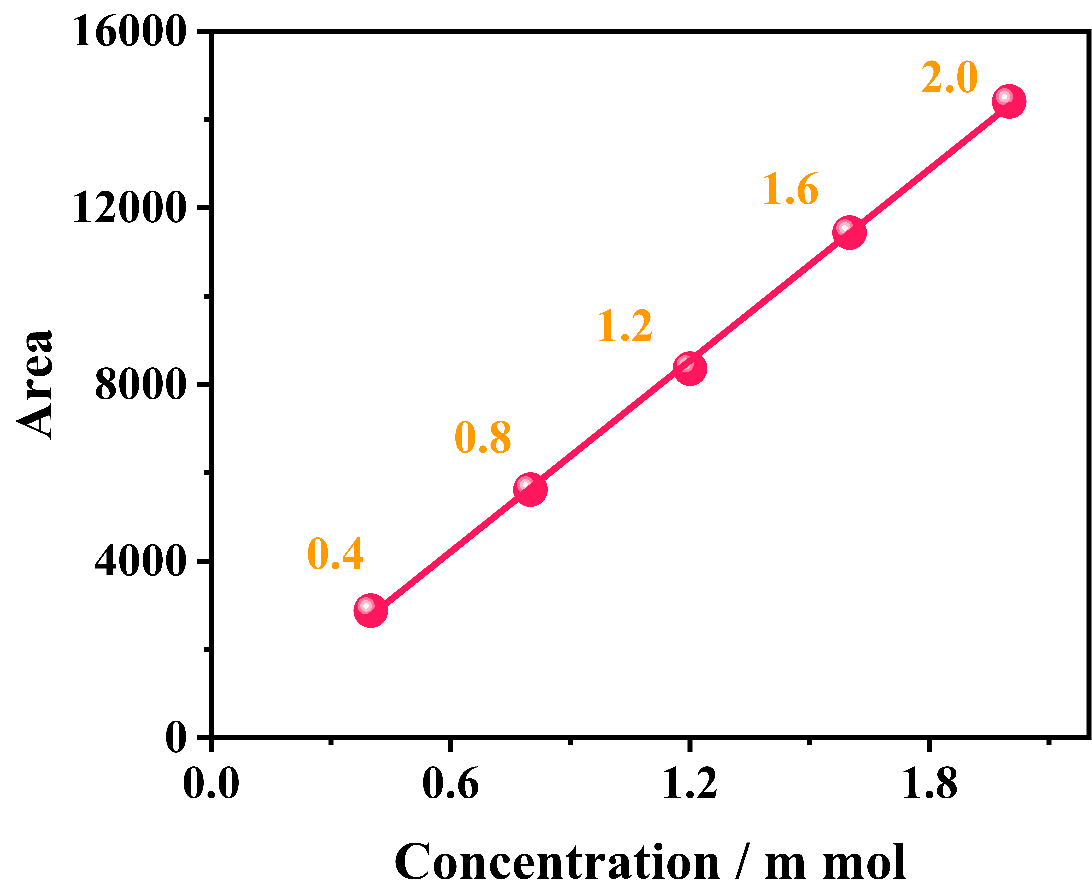
Figure S21.** The standard curves of acetate.

**Figure S22**. ^1^H NMR measurement of the product after 20000 s of the ethanol oxidation on a PdRhFeCoMo HEM electrode at different voltages.

**Figure S23**. ^1^H NMR measurement of the product after 20000 s ethanol oxidation on a) PdFeCoMo MEM and b) PdMo LEM electrodes at different voltages.


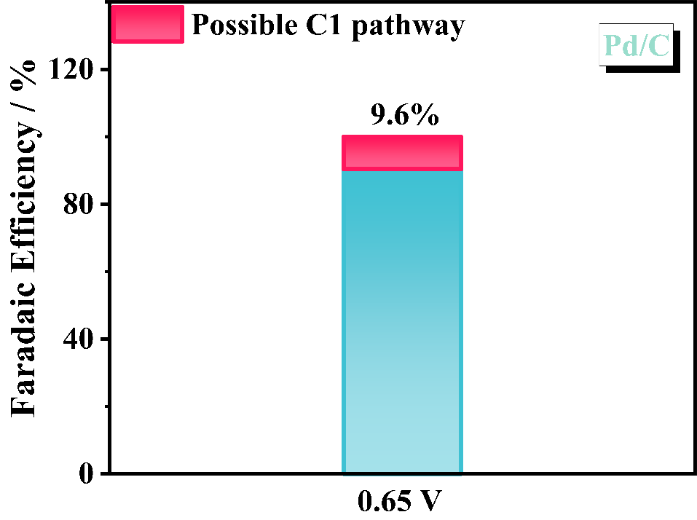


**Figure S24**. Possible C1 pathway of Pd/C.


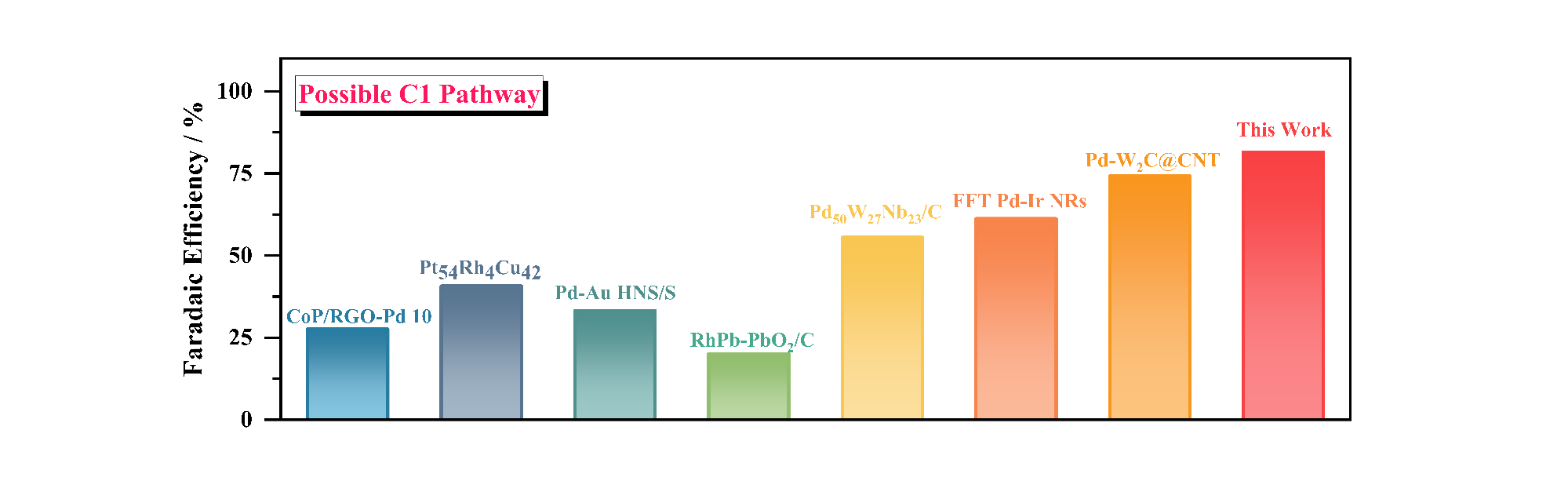
**Figure S25**. Comparisons of C1 selectivity of PdRhFeCoMo HEM with recently reported noble metal-based EOR electrocatalysts.^[12-18]^


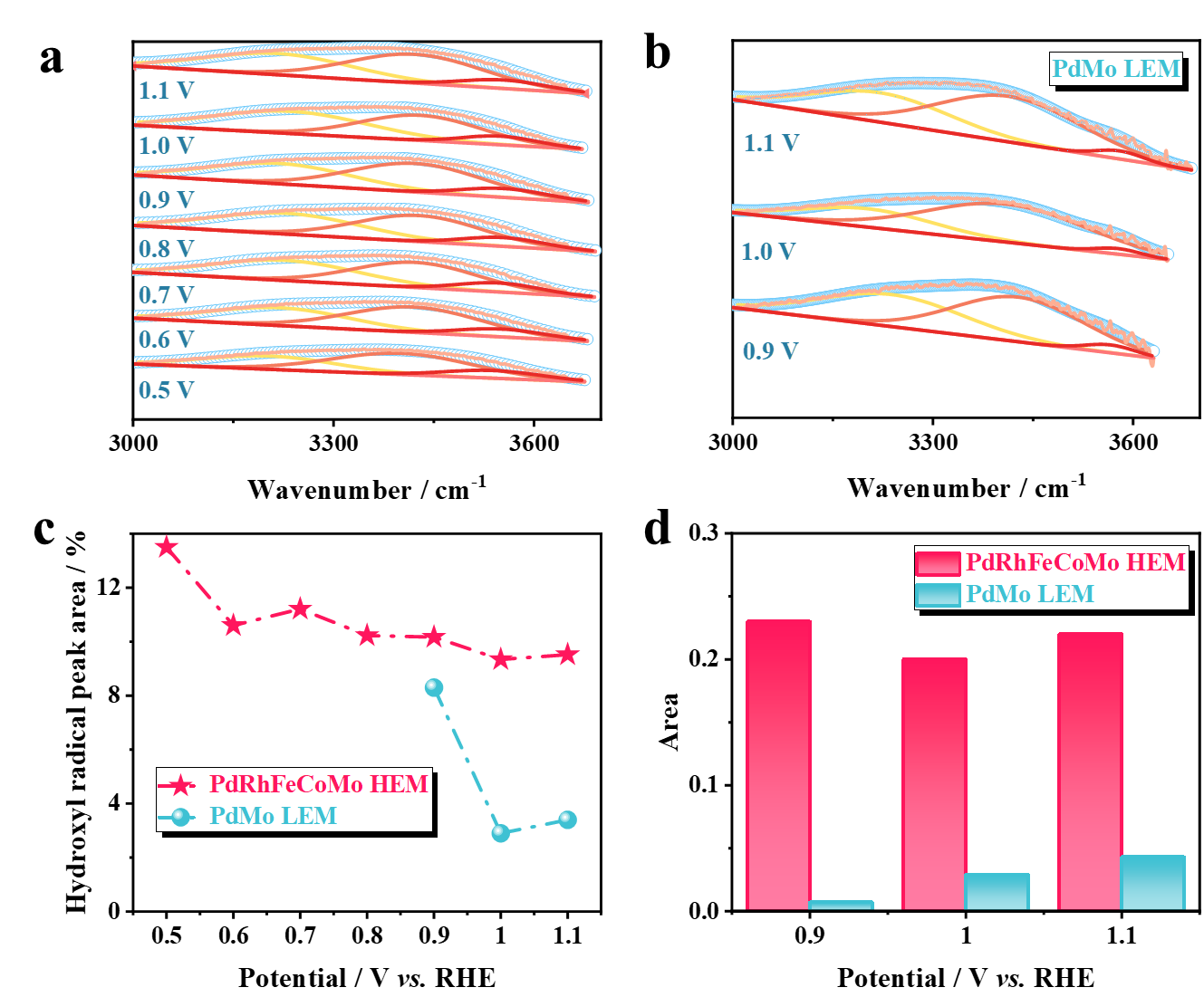


**Figure S26**. The fitted bands at 3000-3700 cm^-1^ of a) PdRhFeCoMo HEM and b) PdMo LEM. c) The integrated absorbance percentage of the OH as a function of the potential, d) Dependence of the integrated absorbance of OH on the potential.


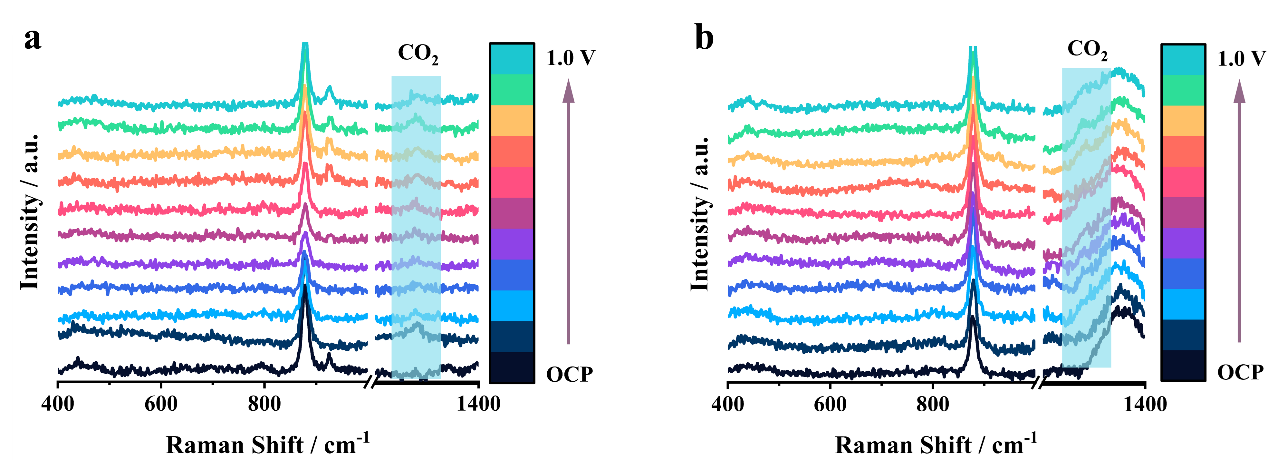


**Figure S27**. Potential-dependent in-situ IERS spectra for a) PdRhFeCoMo HEM and b) PdMo LEM.

**Figure S28**. Raman spectroscopy of electrolyte.
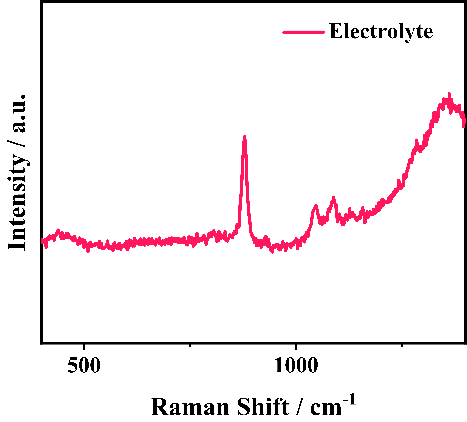


**Figure S29**. PdMo LEM in Ar-saturated 1 M KOH + 1 M CH_3_CH_2_OH electrolyte.


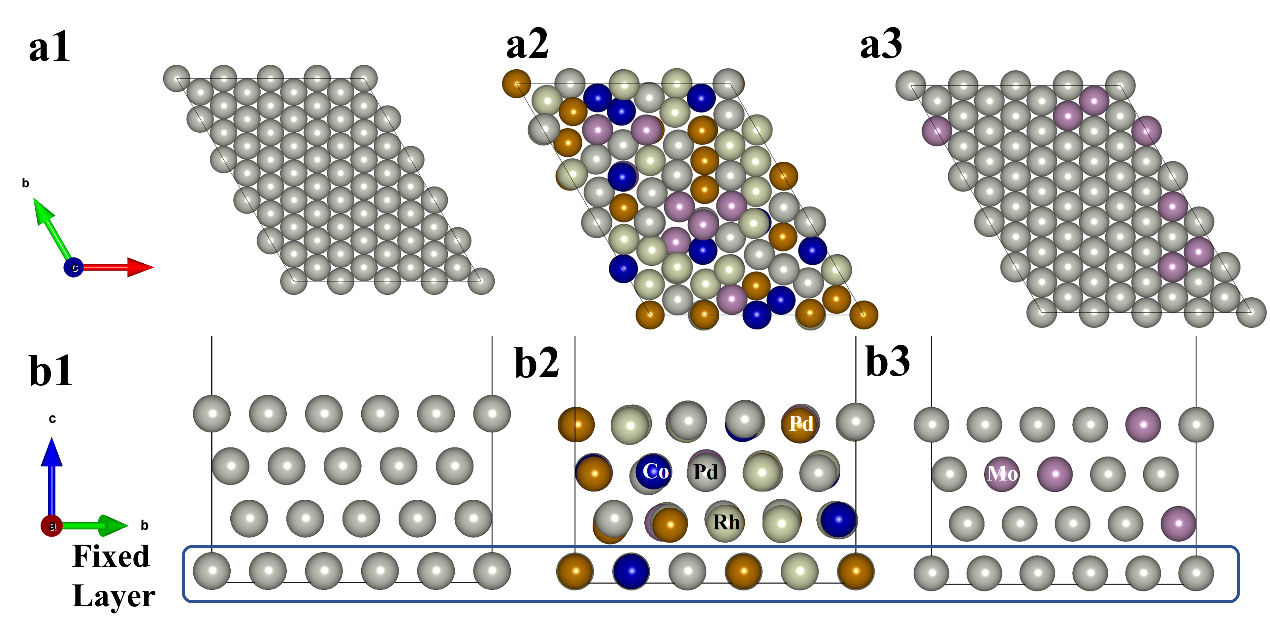


**Figure S30**. Scheme of generating HEM and LEM structures based on the Pd (111) slab model. The top and side view for the structure of Pd (111) (a1, b1), HEM (a2, b2) and LEM (a3, b3).


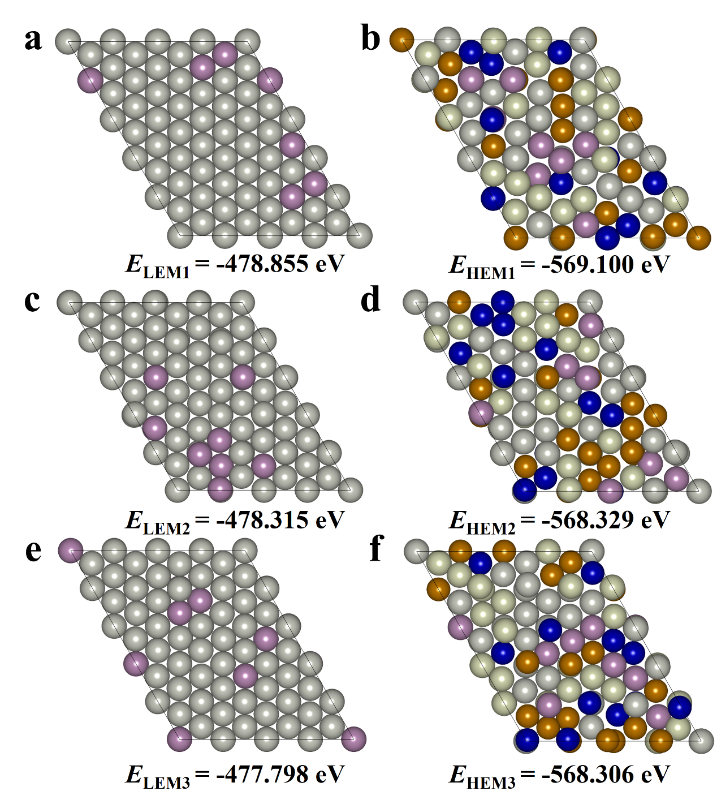


**Figure S31**. The top view for the structure of pre-select structures by ATAT. The energetically favorable model (LEM1 and HEM1) was selected for the whole EOR calculations.


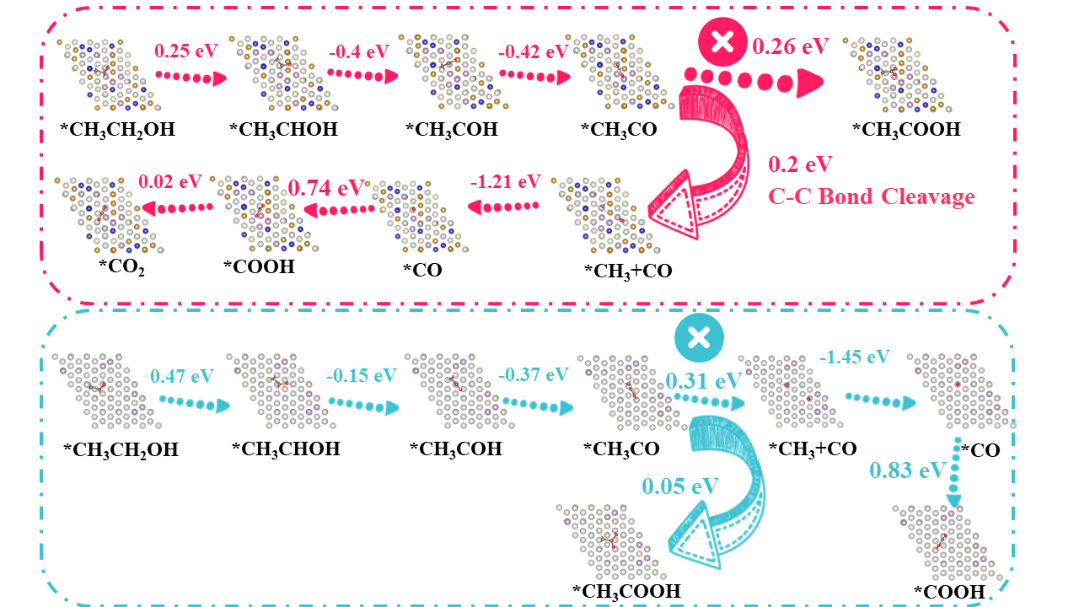
**Figure S32**. Schematic diagram of the pathway selectivity of PdRhFeCoMo HEM and PdMo LEM in EOR.


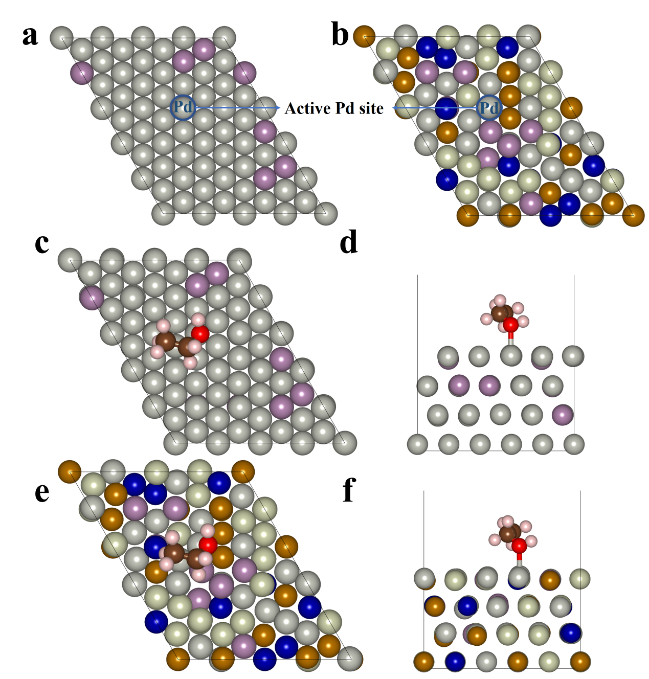


**Figure S33**. The theoretical calculation models of LEM (a) and HEM (b) with the active Pd site noted. The adsorption models for ethanol molecule over LEM (c-d) and HEM (e-f). As has been concluded before, incorporation of palladium significantly enhances the EOR current, thereby substantiating that palladium serves as the active site for the EOR, rather than rhodium (Rh), iron (Fe), cobalt (Co), molybdenum (Mo). Thus, the active Pd site surrounded with other heteroatoms can be regarded as adsorption site.

**
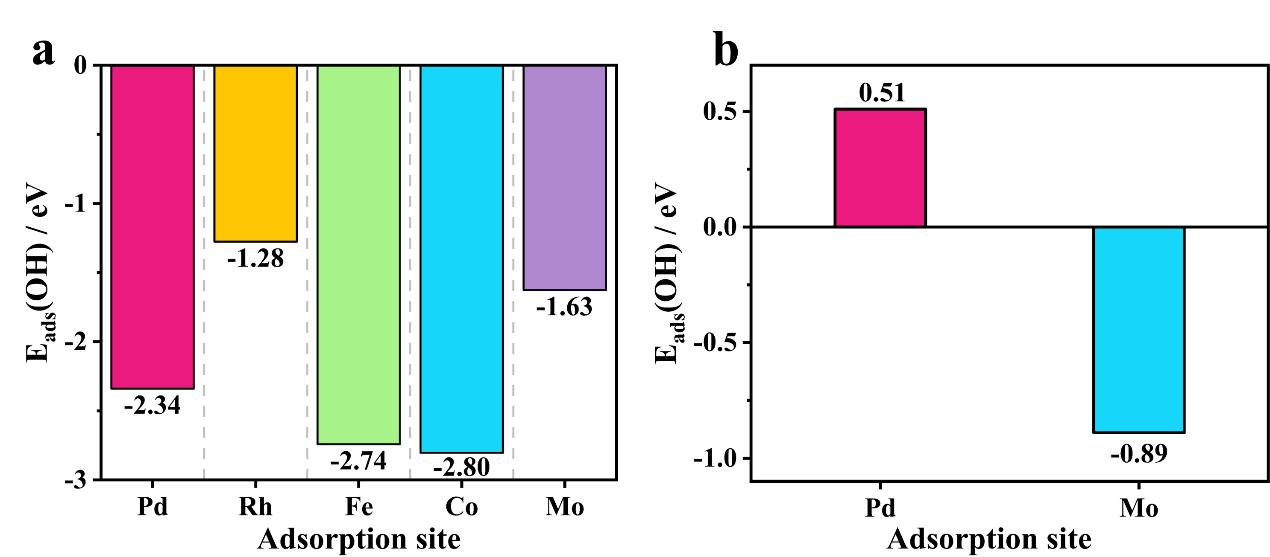
Figure S34**. The adsorption energy of all metals on a) PdRhFeCoMo HEM and b) PdMo LEM for *OH.

**Figure S35**. OCV time plot of DEFCs using PdMo LEM as anode catalyst.

**Figure S36**. DEFCs polarization (left axis) and power-density curves (right axis) using PdMo LEM as anode catalyst.

**Figure S**
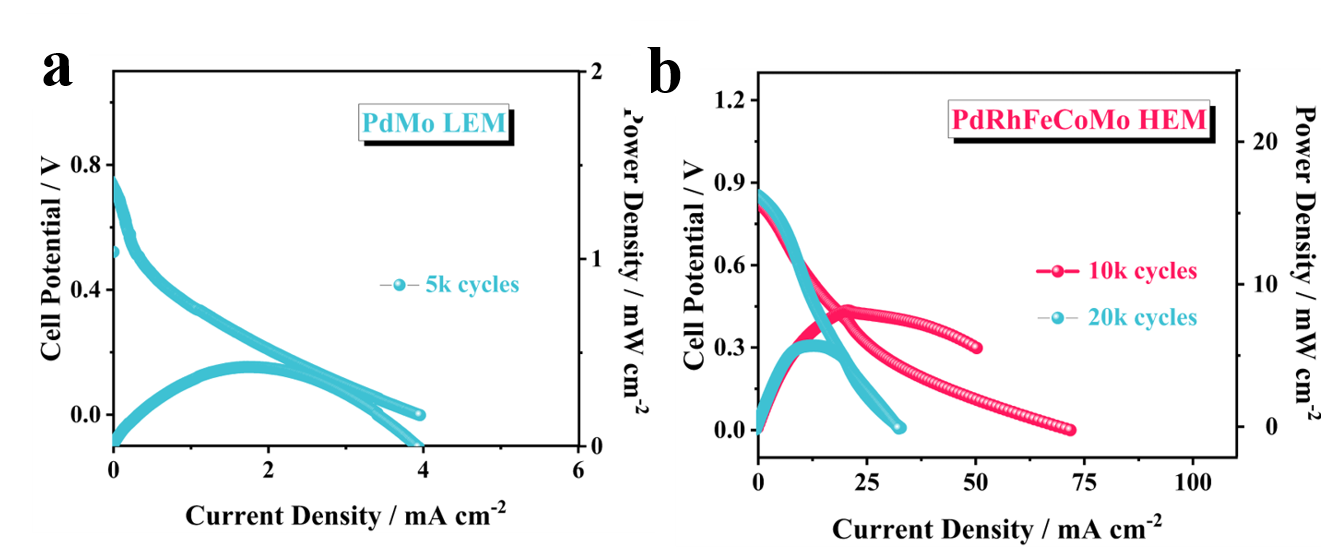
**37.** DEFCs polarization and power density curves of using a) PdMo LEM and b) PdRhFeCoMo HEM as anode catalyst after voltage cycling within 0.6-0.9 V for 5k, 10k, and 20k cycles AST.

***Tables***

**Table S1.** The AFM test results of the three PdRhFeCoMo HEM samples.

| **Sample** | **Thickness (nm)** |
| --- | --- |
| Sample 1 (a) | 1.02 |
| Sample 1 (b) | 1.07 |
| Sample 2 | 1.08 |
| Sample 3 (a) | 1.01 |
| Sample 3 (b) | 1.08 |

**Table S2.** C1 selectivity of PdRhFeCoMo HEM with recently reported noble metal-based EOR electrocatalysts.

| **Catalysts** | **Test Condition** | **Mass Activity**  **A mg_noble metal_^-1^** | **Reference** |
| --- | --- | --- | --- |
| **PdRhFeCoMo HEM** | **1 M KOH+1 M EtOH** | **7.47/9.96** | **This Work** |
| PdAgSn/PtBi HEA NPs | 1 M KOH+1 M EtOH | 3.38 | 19 |
| PtRhBiSnSb | 1 M KOH+1 M EtOH | 15.558 | 20 |
| Pd/Co@N-C | 1 M KOH+1 M EtOH | 7.05 | 21 |
| Rh_7_Bi_1_-NDs | 1 M KOH+0.5 M EtOH | 2.44 | 22 |
| PdCu SMPs | 1 M KOH+1 M EtOH | 6.09 | 23 |
| Pd-Sb RHs/C | 0.5 M NaOH+0.5 M EtOH | 3.48 | 24 |
| Rh-Bi(OH)_3_/C | 1 M NaOH+1 M EtOH | 3.5 | 25 |
| Pd-Au HNS | 1 M KOH+1 M EtOH | 8.0 | 15 |
| HEA-PdPtCuPb UNRs/C | 1 M KOH+1 M EtOH | 18.21 | 26 |
| Au@Pt_1_-Pd_1_ H-Ss | 1 M KOH+1 M EtOH | 3.18 | 27 |
| PdCuBP dMSs | 1 M KOH+1 M EtOH | 5.37 | 28 |
| Pd/a-SrRuO_3_ | 1 M KOH+1 M EtOH | 4.0 | 29 |
| Pt_54_Rh_4_Cu_42_ | 1 M KOH+1 M EtOH | 4.09 | 13 |

**Table S3.** Mass activity and error bar data from multiple tests of different catalysts.

| **Catalysts** | **1** | **2** | **3** | **Aver.**  **(A mg^-1^)** | **Standard Deviation** |
| --- | --- | --- | --- | --- | --- |
| PdRhFeCoMo HEM | 7.75 A mg^-1^ | 7.45 A mg^-1^ | 7.47 A mg^-1^ | 7.55 | 0.16 |
| PdFeCoMo MEM | 5.98 A mg^-1^ | 5.27 A mg^-1^ | 4.08 A mg^-1^ | 5.11 | 0.96 |
| PdMo LEM | 1.49 A mg^-1^ | 1.63 A mg^-1^ | 1.28 A mg^-1^ | 1.47 | 0.17 |

**Table S4.** Comparision of different catalysts regarding the power density and open circuit voltage on DEFCs/DMFCs.

| **Catalysts** | **Power Density**  **(mW cm^-2^)** | **OCP**  **(V)** | **FC Style** | **Reference** |
| --- | --- | --- | --- | --- |
| PdRhFeCoMo HEM | 20.1 | 0.5 | Flexible solid-state DEFCs | This work |
| AlPdNiCuMo | 12.71 | 0.6 | Flexible solid-state DEFCs | *Adv. Funct. Mater.* **2021**, 31, 2007129. |
| AlCuNiPtPdCoMo | 9.2 | 0.8 | Flexible solid-state DEFCs | *Mater. Today Energy* **2021**, 21, 100835. |
| PtMo-CeO_x_-NAs | 15 | ~0.42 | DMFCs | *Chem. Eng. J.* **2022**, 429, 132435. |

**Table S5.** The relevant energies (eV) of adsorbate species during EOR.

| **species** | **CH_3_CHOH** | **CH_3_COH** | **CH_3_CO** | **CH_2_CO** | **COOH** |
| --- | --- | --- | --- | --- | --- |
| ZPE (adsorbed state) | 1.83 | 1.50 | 1.20 | 0.91 | 0.60 |
| **species** | **CH_3_CH2OH** | **CO** | **CH_3_COOH** | **CO_2_** |  |
| ZPE (adsorbed state) | 2.14 | 0.18 | 1.62 | 0.27 |  |
| ZPE (gas state) | 2.10 | 0.13 | 1.61 | 0.31 |  |
| TS (gas state) | 0.87 | 0.61 | 0.88 | 0.66 |  |

**Reference**

1. J. Hafner, *J. Comput. Chem.* **2008**, 29, 2044.
2. G. Kresse, J. Furthmuller, *Phys. Rev. B. Condens. Matter.* **1996**, 54, 11169.
3. J. P. Perdew, Y. Wang, *Phys. Rev. B. Condens. Matter.* **1992**, 45, 13244.
4. J. P. Perdew, J. A. Chevary, S. H. Vosko, A. J. Koblar, R. P. Mark, D. J. Singh, F. Carlos, *Phys. Rev. B. Condens. Matter.* **1992**, 46, 6671.
5. J. P. Perdew, K. Burke, M. Ernzerhof, *Phys. Rev. Lett.* **1996**, *77*, 3865.
6. P. E. Blochl, *Phys. Rev. B. Condens. Matter.* **1994**, 50, 17953.
7. G. Kresse, D.Joubert, *Physical Review B*, **1999**, 59, 1758.
8. S. Grimme, J. Antony, S. Ehrlich, H. Krieg, *J. Chem. Phys.* **2010**, *132*, 154104.
9. A. van de Walle, P. Tiwary, M. de Jong, D. L. Olmsted, M. Asta, A. Dick, D. Shin, Y. Wang, L.-Q. Chen, Z.-K. Liu, *CALPHAD: Computer Coupling of Phase Diagrams and Thermochemistry.* **2013**, 42, 13.
10. A. van de Walle, M. Asta, G. Ceder, *Calphad.* **2003**, 26, 539.
11. Y. Li, W. Nong, Z. Zeng, C. Wang, *Adv. Energy Mater.* **2023**, 13, 2203159.
12. Y. Fang, S. Guo, D. Cao, G. Zhang, Q. Wang, Y. Chen, P. Cui, S. Cheng, W. Zuo, *Nano Res.* **2022**, *15*, 3933.
13. S.-H. Han, H.-M. Liu, P. Chen, J.-X. Jiang, Y. Chen, *Adv. Energy Mater.* **2018**, *8*, 1801326.
14. B. Lan, M. Huang, R.-L. Wei, C.-N. Wang, Q.-L. Wang, Y.-Y. Yang, *Small* **2020**, *16*, 2004380.
15. F. Lv, W. Zhang, M. Sun, F. Lin, T. Wu, P. Zhou, W. Yang, P. Gao, B. Huang, S. Guo, *Adv. Energy Mater.* **2021**, *11*, 2100187.
16. Y. Pan, H. Li, J. Min, J. Xiong, Y. Qin, Z. Wang, Z. Wu, S. Feng, J. Lai, L. Wang, *Chem. Eng. J.* **2022**, *446*, 137178.
17. Y. Qin, H. Huang, W. Yu, H. Zhang, Z. Li, Z. Wang, J. Lai, L. Wang, S. Feng, *Adv. Sci.* **2021**, *9*, 2103722.
18. M. Wang, R. Ding, Y. Xiao, H. Wang, L. Wang, C.-M. Chen, Y. Mu, G.-P. Wu, B. Lv, *ACS Appl. Mater. Interfaces* **2020**, *12*, 28903.
19. X. Lao, X. Liao, C. Chen, J. Wang, L. Yang, Z. Li, J.-W. Ma, A. Fu, H. Gao, P. Guo, *Angew. Chem. Int. Ed.* **2023**, 62, e202304510.
20. W. Chen, S. Luo, M. Sun, X. Wu, Y. Zhou, Y. Liao, M. Tang, X. Fan, B. Huang, Z. Quan, *Adv. Mater.* **2022**, 34, 2206276
21. J. Chang, G. Wang, X. Chang, Z. Yang, H. Wang, B. Li, W. Zhang, L. Kovarik, Y. Du, N. Orlovskaya, B. Xu, G. Wang, Y. Yang, *Nat. Commun.* **2023**, *14*, 1346.
22. B.-Q. Miao, B. Sun, T.-J. Wang, F. Shi, P. Chen, P.-J. Jin, D.-S. Li, F.-M. Li, Y. Chen, *Appl. Catal. B Environ.* **2023**, *337*, 122967.
23. H. Lv, L. Sun, Y. Wang, S. Liu, B. Liu, *Adv. Mater.* **2022**, *34*, 2203612.
24. B. Xu, T. Liu, X. Liang, W. Dou, H. Geng, Z. Yu, Y. Li, Y. Zhang, Q. Shao, J. Fan, X. Huang, *Adv. Mater.* **2022**, *34*, 2206528.
25. B. Lan, Q.-L. Wang, Z.-X. Ma, Y.-J. Wu, X.-L. Jiang, W.-S. Jia, C.-X. Zhou, Y.-Y. Yang, *Appl. Catal. B Environ.* **2022**, *300*, 120728.
26. M. Li, C. Huang, H. Yang, Y. Wang, X. Song, T. Cheng, J. Jiang, Y. Lu, M. Liu, Q. Yuan, Z. Ye, Z. Hu, H. Huang, *ACS Nano* **2023**, 17, 13659.
27. W. Liang, Y. Wang, L. Zhao, W. Guo, D. Li, W. Qin, H. Wu, Y. Sun, L. Jiang, *Adv. Mater.* **2021**, *33*, 2100713.
28. H. Lv, Y. Wang, D. Xu, B. Liu, *Nano Res.* **2021**, *14*, 3274.
29. X. Wu, J. He, M. Zhang, Z. Liu, S. Zhang, Y. Zhao, T. Li, F. Zhang, Z. Peng, N. Cheng, J. Zhang, X. Wen, Y. Xie, H. Tian, L. Cao, L. Bi, Y. Du, H. Zhang, J. Cheng, X. An, Y. Lei, H. Shen, J. Gan, X. Zu, S. Li, L. Qiao, *Nano Energy* **2020**, *67*, 104247.
